# Inorganic fertilizer use and its association with rice yield gaps in sub-Saharan Africa

**DOI:** 10.1016/j.gfs.2023.100708

**Published:** 2023-09

**Authors:** Jean-Martial Johnson, Ali Ibrahim, Elliott Ronald Dossou-Yovo, Kalimuthu Senthilkumar, Yasuhiro Tsujimoto, Hidetoshi Asai, Kazuki Saito

**Affiliations:** a Africa Rice Center (AfricaRice), 01 B.P. 2551, Bouaké 01, Cote d'Ivoire; b Institute of Crop Science and Resource Conservation (INRES), University of Bonn, D-53115, Bonn, Germany; c Africa Rice Center (AfricaRice), PMB 82, 901101, Abuja, Nigeria; d Africa Rice Center (AfricaRice), B.P. 1690, Antananarivo 101, Madagascar; e Japan International Research Center for Agricultural Sciences (JIRCAS), 1-1 Ohwashi, Tsukuba, Ibaraki, 3058686, Japan; f International Rice Research Institute (IRRI), DAPO Box 7777, Metro Manila 1301, Philippines

**Keywords:** Agroecological zone, Fertilizer use efficiency, *Oryza* spp., Partial factor productivity, Yield gap

## Abstract

Where and which countries should receive higher priority for improving inorganic fertilizer use in rice fields in sub-Saharan Africa (SSA)? This study addressed this question by assessing the spatial variation in fertilizer use and its association with rice yield and yield gap in 24 SSA countries through a systematic literature review of peer-reviewed papers, theses, and grey literature published between 1995 and 2021. The results showed a large variation in N, P, and K fertilizer application rates and rice yield and an opportunity for narrowing the yield gap by increasing N and P rates, especially in irrigated rice systems. We identified clusters of sites/countries based on nutrient input and yield and suggested research and development strategies for improving yields and optimizing nutrient use efficiencies. Further research is essential to identify the factors causing low fertilizer use and the poor association between its use and yield in rainfed systems.

## Highlights

• We reviewed inorganic fertilizer use and rice yield in 24 sub-Saharan African countries.
• N and P fertilizer rates were higher in irrigated lowlands and arid zone.
• Higher N and P rates were associated with a lower yield gap in irrigated rice systems.
• Higher P application rates improved the partial factor productivity of applied N.
• About 40% of the data points showed a high risk of soil nutrient mining.

## Graphical abstract

**Figure:**
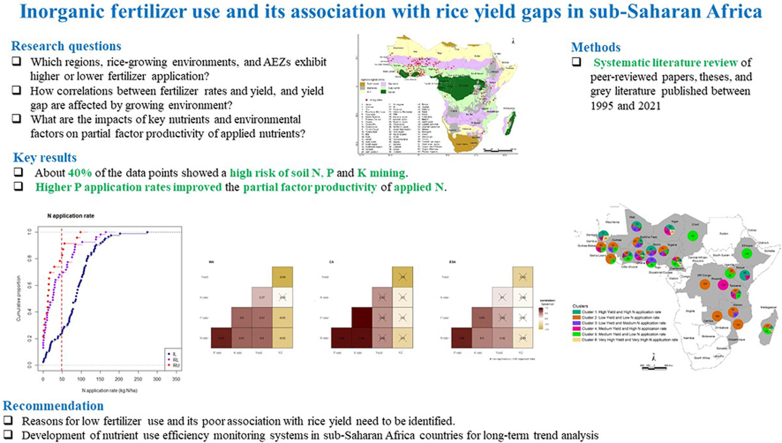

## 1 Introduction

Inorganic fertilizers (hereafter referred to as fertilizers) have strongly contributed to the agricultural production boom of the 1960s and 70s known as the “Green Revolution” in America and Asia. In production systems of major staple foods such as rice (*Oryza* spp.), fertilizer application is one of the key drivers for improving productivity (Gu and Yang, 2022) thereby keeping pace with the fast-growing human population. When the application rates are too low, there are high risks of soil mining and lower productivity with detrimental effects on food security. Very low fertilizer application rate and poor soil fertility management, causing low productivity and rapid soil depletion also lead to crop area expansion with its corollaries that are massive biodiversity loss, higher greenhouse gas (mainly CO_2_) emissions from tropical deforestation, soil, and land degradation, siltation of water courses and reservoirs, and increasing human conflicts (Scientific Panel on Responsible Plant Nutrition, 2021). Conversely, excessive use leads to nutrient losses that cause degradation of downstream water quality, eutrophication of freshwater, rising nitrous oxide (N_2_O) emissions (Vitousek et al., 1997), and reduction of benefit-cost ratio.

Unlike most regions of the world, crop yields have not increased substantially in sub-Saharan Africa (SSA), and as a consequence, a large share of its population faces hunger (Arouna et al., 2021b; FAO et al., 2022). Being a traditional staple food in many parts of Africa, especially in the West Africa region, and the second most important source of calories in SSA (Carneyl, 1998; Seck et al., 2013), rice plays a major role in achieving food security. However, despite the release of high-yielding rice cultivars (Futakuchi et al., 2021) and the development of improved crop and water management practices (Dossou-Yovo et al., 2022; Senthilkumar, 2022), rice productivity is still low in SSA, with mean yields of 2.2 Mg ha^−1^ compared to mean yields of 4.8 Mg ha^−1^ and 6.1 Mg ha^−1^ reported in Asia and South America, respectively (FAO, 2021). The average yield gap (i.e., the difference between the potential yield in irrigated lowland or water-limited yield in rainfed systems and the actual yield obtained by farmers) is large and estimated at around 5.0 Mg ha^−1^ in irrigated lowland, 5.3 Mg ha^−1^ in rainfed lowland and 5.6 Mg ha^−1^ in rainfed upland (Dossou-Yovo et al., 2020). Furthermore, past studies have consistently stressed that inadequate and low fertilizer inputs and poor soil fertility are major limiting factors to rice production in SSA (Haefele et al., 2013; Tsujimoto et al., 2019) and that higher rice yield was associated with higher N application rate (Ibrahim and Saito, 2022).

Information on yield gaps can help identify regions with the greatest potential to increase current yield through the use of cost-effective agronomic practices and inputs. Likewise, quantifying fertilizer application rate at national, and regional levels is an essential component of fertilizer consumption analysis and demand projection as fertilizers often represent the major source of nutrient input in cropping systems (Vitousek et al., 2009).

Although the rice yield gap in SSA has been analyzed in detail (Senthilkumar et al., 2020; van Oort et al., 2015, 2017), insufficient attention was paid to the spatial variation of N, P, and K fertilizer application at the continental scale. Past studies have evidenced that inorganic fertilizers are powerful productivity-enhancing inputs in rice fields in SSA (Awio et al., 2021; Bado et al., 2018; Saito et al., 2019). To date, relatively little research has analyzed the correlation between nutrients applied through inorganic fertilizers by smallholder farmers and the rice yield and yield gap in SSA (Arouna et al., 2021a; Dossou-Yovo et al., 2020; Ibrahim et al., 2022). The recently released datasets on Fertilizer Use by Crop and Country (FUBC) contained data for rice from just four countries in SSA (Ludemann et al., 2022). A comprehensive and African continental-wide analysis of the past three decades is missing. Therefore, we conducted a systematic literature review of fertilizer use in smallholder rice farmers’ fields aiming to investigate the spatial variation in fertilizer use and assess its relationship with yield and yield gap in different growing environments in SSA. This study set out to answer the following questions: (i) Which regions, rice-growing environments, and agroecological zones exhibit higher or lower fertilizer application? (ii) How the correlations between fertilizer rates and yield, yield gap, and partial factor productivity of applied nutrients are affected by the growing environment? (iii) What are the impacts of key nutrients and environmental factors, such as agroecological zones and growing environments on the partial factor productivity of applied nutrients? This overview is of high importance, on one hand, for scientists, extensionists, and industrials as it provides relevant information on nutrients partial factor productivity and could help in forecasting future demand. On the other hand, it could guide international agencies and governments in rice research and development prioritization, and specifically to know where new investments can achieve the highest impact.

## 2 Methods

### 2.1 Search strategy, selection criteria, and database compilation

We did a systematic literature review and collected data on inorganic fertilizer use in rice fields in sub-Saharan Africa from different research engines; the main one being Google Scholar (https://scholar.google.com/). The searches have been done in English and French to identify studies published before August 20th, 2021 (the end date of our search). By combining the research terms as follow: “fertili*” AND “rice” AND “Africa” OR “NPK” OR “Urea” OR “fertilizer use efficiency” OR “paddy” OR “*Oryza*”, we compiled all research papers including original peer-reviewed papers, theses, and grey literature (research, technical, and project reports, working papers, government documents). To ensure comparability, the inclusion or the exclusion criteria were as follows: (i) survey or farmers' field trials carried out in smallholder farmers' fields in SSA where fertilizer management (fertilizer type, application time and rates) was done according to local practices were included; (ii) studies reporting data on recommended application rates from research-managed trials conducted in research station or farmers' fields were excluded because they were considered not representative of farmers’ practices; (iii) studies reporting pot experiments were also excluded; (iv) Both grain yield data and fertilizer (N, P, and K) application rates should be provided. Then, we removed duplicate studies and irrelevant literature. In addition, for Burkina Faso and Malawi, we retrieved and aggregated agronomic data from the national agricultural statistics dashboard (Ministère de l’Agriculture et des Aménagements Hydro-agricoles, 2021) and the World Bank Living Standards Measurement Study-Integrated Surveys on Agriculture (LSMS-ISA) initiative (Kilic et al., 2015; World Bank, 2021). Non-fertilized fields (i.e., zero input) were included in the calculation of the average nutrient application rates. We also retrieved data from the Fertilizer Use by Crop and Country (FUBC) (Ludemann et al., 2022) and the International Fertilizer Development Center (IFDC) (IFA/IFDC/IPI/PPI/FAO, 2002). As grain yield data was missing for these data points, we matched the fertilizer application rate data at the national level with the grain yield data for the same year from FAOSTAT dataset (FAO, 2021). Finally, we requested colleagues (rice scientists working in SSA countries) to provide unpublished data and received 5 aggregated data (or 5 data points) from surveys carried out between 2019 and 2020 in four sites in Côte d’Ivoire.

Data on the following study variables were extracted using a predefined form: country, sub-national and location name, year, season (wet or dry), growing environments (irrigated lowland, rainfed lowland, or rainfed upland), N, P, and K application rates, typical N fertilizer, typical compound fertilizer, and grain yield. The dataset was enriched with information on the geographic position (i.e., coastal or landlocked country), region (i.e., West Africa, Central Africa, or East and Southern Africa), agroecological zone (humid, sub-humid, semi-arid, arid or highlands) (HarvestChoice and International Food Policy Research Institute, 2015). We did not attribute an agroecological zone (AEZ) to data points (or observations) at the country level. For each site, whenever available in a study, we retrieved the potential yield in irrigated lowland systems or the water-limited potential yield in rainfed systems. If not, we completed the missing information with the data from the Global Yield Gap Atlas (GYGA) website (https://www.yieldgap.org/*(**GYGA team, 2022**)*.

The final database consists of 235 data points (or observations) from 1995 to 2021 (collected from 23 published papers or datasets and 2 unpublished) (See Supplemental material). Here, a data point is the unit of analysis and refers to average data (fertilizer rate or yield) by site or country * season and/or year. To ensure data quality, we performed Rosner's test to identify outliers for N, P, K rates, and grain yield and removed extreme outliers [i.e., values above Q3 (third quartile) + 3 IQR (interquartile range)] *(**Kassambara, 2023*; *Rosner, 1983**)*.

### 2.2 Calculations

For each data point (or observation), the partial factor productivity (PFP) of a given nutrient N (PFPN), P (PFPP), or K (PFPK) was calculated to show the grain yield (in kg grain ha^−1^) per unit of nutrient *x* applied (kg *x* ha^−1^). N, P, and K values are expressed on an elemental basis. Thus, PFP (kg grain kg^−1^) was calculated according to Equation (1).

(1) $$PFPx=\frac{Grain\;yield}{Rate\;of\;nutrient\;x\;applied}$$

The absolute yield gap was calculated as the difference between the potential yield (in irrigated lowland) or water-limited potential yield (in rainfed systems) (Yw) and the average farmers’ grain yield (Ya). The relative yield gap (YG) was calculated as the ratio between the absolute yield gap and the potential yield (in irrigated lowland) or water-limited potential yield (in rainfed systems) (Yw) and expressed in percentage (Equation (2)).

(2) $$YG=\frac{Yw-Ya}{Yw}\times 100$$

### 2.3 Statistical analysis

Descriptive statistics (mean and standard deviation, median and interquartile range) were used to characterize fertilizer (N, P, and K) application rates, grain yield, partial factor productivity (PFP), and relative yield gap at the country, geographic position, region, growing environment or AEZ levels. Differences between regions, AEZs, and growing environments for nutrient application rates, grain yield, partial factor of productivity (PFP), and relative yield gap were assessed using one-way analysis of variance (ANOVA) when data met the requested assumptions (i.e., normality of residuals and homoscedasticity) and if not using non-parametric Kruskal-Wallis test. Wherever the ANOVA or the Kruskal-Wallis test were significant (p-value ≤0.05), post hoc tests (Tukey honestly significant difference test for ANOVA and Dunn test for Kruskal-Wallis test) were performed for multiple comparisons of groups. For factors having only two modalities such as geographic position (coastal countries vs landlocked countries) and whose data did not follow a normal distribution, we assessed the difference using a Wilcoxon-Mann-Whitney test (Mangiafico, 2016).

The benchmark values for PFPN, PFPP, and PFPK were customized for SSA countries according to Arouna et al. (2021a), and three categories were defined. Optimal PFP is met when recommended management practices are followed. The category “Very low” corresponds to an over-application and a high risk of wasteful application while “Very high” suggests a high risk of soil nutrient mining (Fixen et al., 2015). We determined the share of data points presenting optimum PFPN, PFPP, and PFPK. To determine the probabilities of belonging to one of the categories of PFPN, PFPP, and PFPK, we used multinomial logit (MNL) models. The MNL regressions were run for PFPN, PFPP, and then PFPK using the *nnet* package (Venables and Ripley, 2002) with five predictor variables: N, P, and K application rates, AEZs and growing environments.

Correlation analyses were performed to examine the relationship between N, P, and K application rates, yield, and relative yield gap (YG) across growing environments. The pairwise association between variables was visualized using a correlogram. As the standard Pearson's correlation estimate is parametric (i.e., based on normality and homoscedasticity assumptions) and can be heavily influenced by extreme values, we either computed the Spearman's rank correlation coefficient (ρ) for mitigating the outliers' concerns (Wilcox, 2016). The magnitude of the effect size ρ was interpreted according to Cohen (1992).

We conducted an in-depth analysis to assess the scope to increase actual yields by increasing N input and thereby identify priority intervention countries, regions, or growing environments. Thus, we performed a hierarchical agglomerative cluster analysis (Kassambara and Mundt, 2020) using Euclidean distance and following Ward's method (Ward, 1963) on 224 data points using two variables (N application rate and grain yield). According to the Elbow method (Kassambara and Mundt, 2020), the data points were classified into six distinct clusters. We constructed pie charts of this clustering per country and displayed results on a map at the country level as the number of data points for some sites was too small to provide meaningful information at the sub-national level. All the statistical analyses were done using R software, Version 4.2.3 (R Core Team, 2023).

## 3 Results

**Fig. 1:**
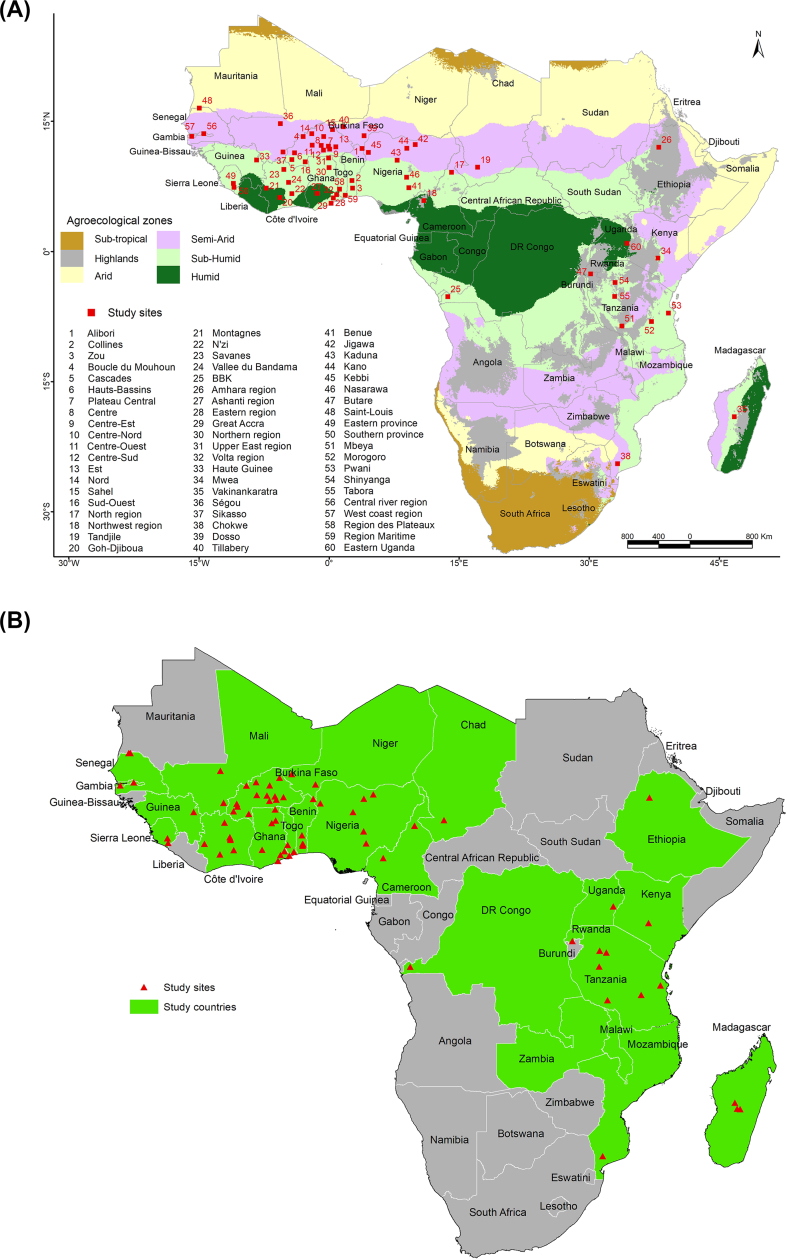
Location of the study sites (A) across five agroecological zones (Humid, Sub-Humid, Semi-Arid, Arid, and Highlands) and (B) in twenty-four sub-Saharan African countries.

Our review yielded 235 data points from studies conducted between 1995 and 2020 in the three SSA regions: West Africa (171 data points), Central Africa (9 data points), and East and Southern Africa (55 data points). The data points were distributed in three rice-growing environments [irrigated lowland (86 data points), rainfed lowland (55 data points), and upland (24 data points)], five agroecological zones [Humid (14 data points), Sub-Humid (76 data points), Semi-arid (48 data points), Arid (13 data points), and Highlands (12 data points)] (Fig. 1A) and across 24 sub-Saharan African countries (Fig. 1B). Growing environment and AEZ information were missing for 70 and 72 observations, respectively. Sorted by descending order, Burkina Faso, Senegal, Nigeria, Côte d’Ivoire, and Ghana were the countries having more data points (Fig. S1) and gathered together half (51%) of the data points.

### 3.1 Fertilizer application rate and partial factor productivity of N, P, and K

**Fig. 2:**
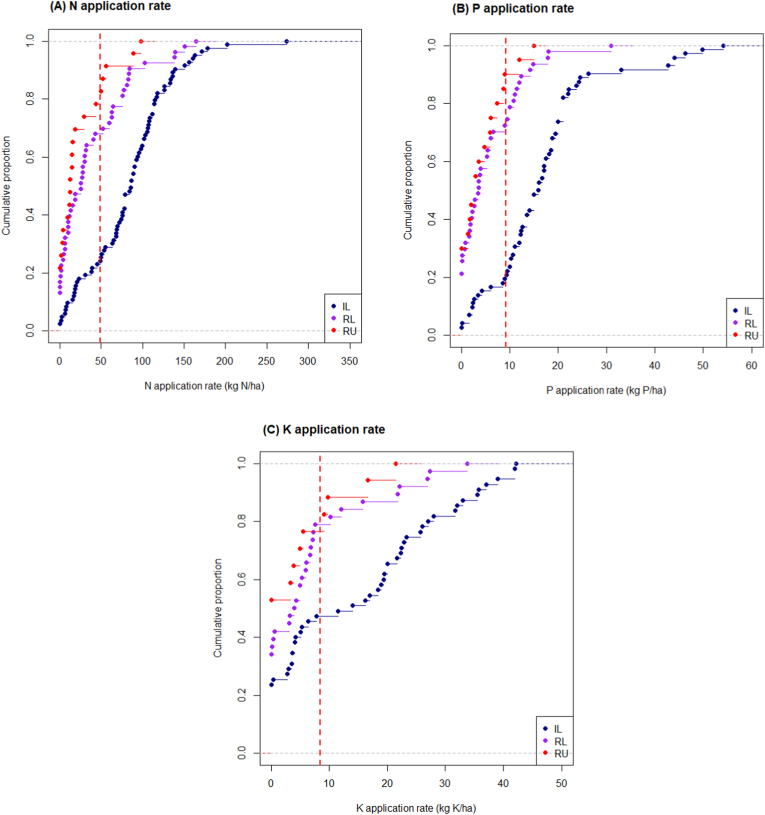
Cumulative distribution probability of (A) N, (B) P, and (C) K fertilizer application rates (kg ha^−1^) from data points in irrigated lowland (IL), rainfed lowland (RL), and, rainfed upland (RU) retrieved from surveys/studies conducted in sub-Saharan Africa between 1995 and 2020. The dashed vertical red lines indicate the global average application rate for the corresponding nutrient.

The N, P, and K fertilizer application rates largely varied (CV ranging between 111 and 130%) with an average of 49, 9, and 8 kg ha^−1^, respectively. About 8%, 11%, and 27% of data points reported no application of N, P, and K fertilizers (i.e., 0 kg ha^−1^), respectively. Table S1 presents the average N, P, and K fertilizer application rates across countries and growing environments in SSA. The geographic position of a country (i.e., coastal or landlocked country) has no significant effect on the fertilizer application rates (Table S2). However, a spatial variation was visible across regions, agroecological zones, and growing environments. For example, on average, N, P, and K fertilizer application rates were higher in West Africa (WA) (58 kg N ha^−1^, 11 kg P ha^−1,^ and 11 kg K ha^−1^) than in Central Africa (CA) (31 kg N ha^−1^, 3 kg P ha^−1^ and 4 kg K ha^−1^) and East and Southern Africa (ESA) (25 kg N ha^−1^, 5 kg P ha^−1^ and 1 kg K ha^−1^) (Table S3). Farmers applied more N, P, and K fertilizers in irrigated lowlands (84 kg N ha^−1^, 17 kg P ha^−1^, and 15 kg K ha^−1^) than in rainfed systems (Table S4). About 23%, 68%, and 78% of data points from irrigated lowland, rainfed lowland, and upland had an N application rate lower than the average in SSA (49 kg N ha^−1^), respectively (Fig. 2A). For P application, about 19%, 70%, and 85% of data points from irrigated lowland, rainfed lowland, and upland had an application rate lower than the average in SSA (9 kg P ha^−1^), respectively (Fig. 2B). And for K application, about 47%, 79%, and 76% of data points from irrigated lowland, rainfed lowland, and upland had a lower application rate than the average in SSA (8 kg K ha^−1^), respectively (Fig. 2C). In arid zone, N and P application rates (139 kg N ha^−1^ and 18 kg P ha^−1^) were higher than in humid, sub-humid and highlands zones (Table S5).

Partial factor productivity of nutrients largely varied (CV ranging from 186 to 521%) with PFPP having the highest coefficient of variation (521%). Furthermore, significant differences were observed among regions but not among growing environments and AEZs (Tables S3, S4, and S5). Overall, 48%, 41%, and 54% of the data points were within the optimum range of PFPN, PFPP, and PFPK, respectively. About 40% of the data points had very high partial factor productivity (PFP) of N, P, and K compared to the optimum level, implying an insufficient supply of inorganic fertilizer and a high risk of soil nutrient mining (Tables S6, S7, and S8). The proportion of data points having a “Very high” PFPN was higher in rainfed systems than in irrigated lowlands while rainfed lowlands had the highest proportion of data points in the category “Very high” of PFPP and PFPK (Figs. S2A, S2B, and S2C). In CA and ESA, 67% and 62% of data points, respectively, were categorized as “Very high” PFPN. Conversely, in WA, only 29% of data points were categorized as “Very high” PFPN and more than half of the data points were in the optimum range of PFPN. Similar trends were found for PFPP and PFPK (Table S6).

### 3.2 Grain yield and yield gap

On average, grain yield was 2.98 Mg ha^−1^ across all sites, with a 57% coefficient of variation. The variability was higher in rainfed lowlands (53%) than in uplands (34%) and irrigated lowlands (28%). There was a significant difference in grain yield among the growing environments and AEZs (Tables S4 and S5) but not among the regions (Table S3). The mean yields were 4.56, 2.54, and 1.69 Mg ha^−1^ in irrigated lowland, rainfed lowland, and upland (Table S4). About 88%, 29%, and 0% of data points from irrigated lowland, rainfed lowland, and upland had a higher grain yield than the global average yield (2.98 Mg ha^−1^), respectively (Fig. S3).

On average, the relative yield gap was 56% with a significant difference between growing environments and AEZs. It was lower in irrigated lowlands (46%) than in rainfed lowlands (66%) and uplands (74%) (Table S4). Among AEZ, the relative yield gap was the lowest in the Arid zone (27%) (Table S5).

### 3.3 Relationship between nutrients application rate, grain yield, and relative yield gap

**Fig. 3:**
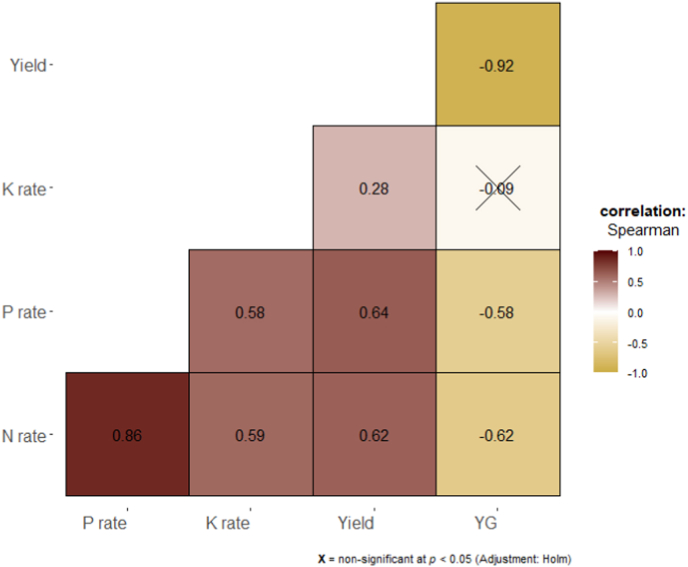
Correlogram showing the relationships between nutrients (N, P, and K) application rates, rice grain yield, and relative yield gap in different studies in sub-Saharan Africa. The values displayed in the matrix are Spearman's rank correlation coefficients (ρ). Non-significant ρ (*p* < 0.05) are crossed out. Positive correlations are displayed in maroon (or dark red) and negative correlations in goldenrod. The color intensity (see color bar) of the square is proportional to the correlation coefficient.

**Fig. 4:**
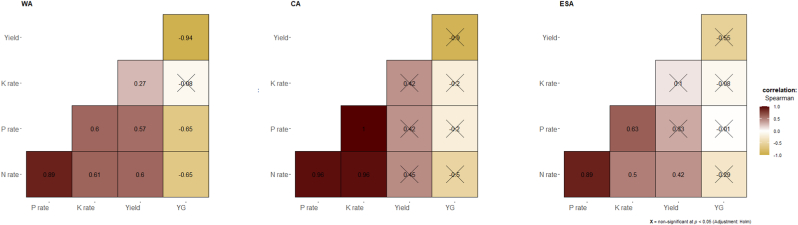
Correlogram showing the relationships between nutrients (N, P, and K) application rates, rice grain yield, and relative yield gap in different studies across regions [West Africa (WA), Central Africa (CA), and, East and Southern Africa (ESA)] in sub-Saharan Africa. The values displayed in the matrix are Spearman's rank correlation coefficients (ρ). Non-significant ρ (*p* < 0.05) are crossed out. Positive correlations are displayed in maroon (or dark red) and negative correlations in goldenrod. The color intensity (see color bar) of the square is proportional to the correlation coefficient.

**Fig. 5:**
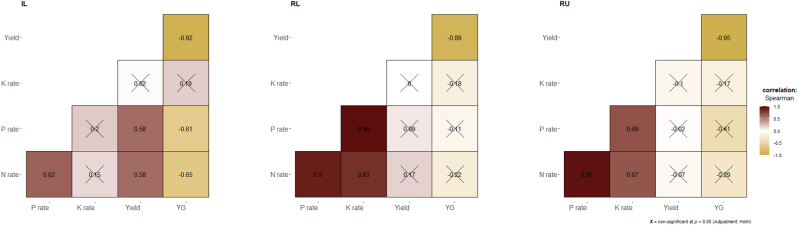
Correlogram showing the relationships between nutrients (N, P, and K) application rates, rice grain yield, and relative yield gap in different studies across growing environments [irrigated lowland (IL), rainfed lowland (RL), and, rainfed upland (RU)] in sub-Saharan Africa. The values displayed in the matrix are Spearman's rank correlation coefficients (ρ). Non-significant ρ (*p* < 0.05) are crossed out. Positive correlations are displayed in maroon (or dark red) and negative correlations in goldenrod. The color intensity (see color bar) of the square is proportional to the correlation coefficient.

Overall, N and P application rates had a strong effect (ρ = 0.6) on rice yield and the reduction of the relative yield gap (ρ = −0.6) while the K application rate had a small effect (ρ = 0.28) on yield and no effect on the reduction of the yield gap (Fig. 3). These correlations among N, P, and K application rates, grain yield, and relative yield gap varied among regions (Fig. 4), growing environments (Fig. 5), and AEZs (Fig. S4). For example, in WA, N and P application rates had a strong effect (ρ = 0.6) on rice yield and the reduction of the relative yield gap (ρ = −0.6), while in ESA, only the N application rate had a medium effect (ρ = 0.4) on yield. Except in WA where the K application rate had a small effect (ρ = 0.27) on yield, no effect was obvious in CA and ESA (Fig. 4). Among growing environments, in irrigated lowland, N and P application rates had a strong effect (ρ = 0.6) on rice yield and reduction of the relative yield gap (ρ = −0.6), but no effect was obvious in rainfed systems (Fig. 5).

### 3.4 Impacts of nutrients application rate and environment on partial factor productivity of N, P, and K

**Fig. 6:**
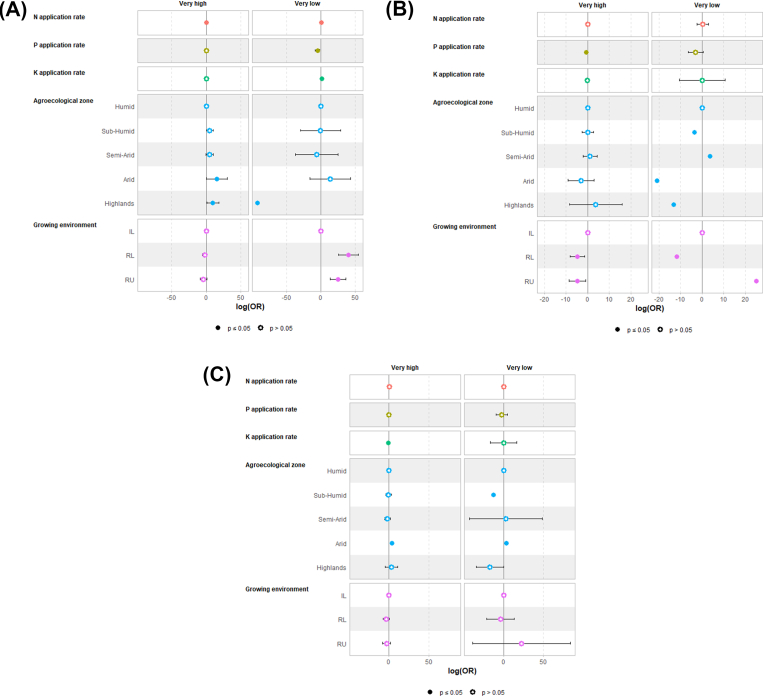
Estimated parameters of multinomial logistic regression: effects of the nutrients application rates and the environmental conditions on the optimum levels of (A) PFPN, (B) PFPP, and (C) PFPK in different studies in sub-Saharan Africa. Empty dot symbols mean the regression coefficient is not significant (p > 0.05). The filled dot symbol means the regression coefficient is significant (p < 0.05).

Nutrients application rates and growing environments had variable effects on the partial factor productivity of N, P, and K. Concerning PFPN, reducing the N application rate raised the likelihood of being classified in the category “Very high” while increasing the rate raised the likelihood of being classified in the category “Very low”. A lower P application in fields was associated with a higher probability of “Very low” PFPN. In comparison with farmers’ fields in the Humid zone, the ones from the Arid and Highlands zones were more likely to be categorized in the group “Very high”. In comparison with fields in irrigated lowlands, growing rice in rainfed systems raised the likelihood of having a “Very low” PFPN or in other words wasteful application of N (Fig. 6A). A lower P application was associated with a higher probability of “Very high” PFPP. In comparison with fields in irrigated lowlands, growing rice in rainfed systems reduced the likelihood of having a “Very high” PFPP i.e., of having a risk of soil P mining. Fields in rainfed uplands had a higher probability to be classified in the category “Very low” (Fig. 6B). Concerning PFPK, reducing the K application rate raised the likelihood of being classified in the category “Very high” (Fig. 6C).

### 3.5 Cluster analysis and priority intervention areas

Six distinct clusters were identified (Fig. S5). Cluster 6 (VHYVHN) has a very high yield (mean = 6.69 Mg ha^−1^) and a very high N application rate (mean = 171 kg N ha^−1^). The PFPN for this cluster is on average 42 kg grain kg^−1^ N. Around 4% of the data points belong to this cluster. Accounting for 20% and 12% of the data points respectively, cluster 1 (HYHN) and cluster 4 (MYHN) have high N application rates (mean 93 and 87 kg N ha^−1^, respectively). They differ in their actual yield. Cluster 1 has high yields (mean 5.16 Mg ha^−1^) while cluster 4 has medium yields (mean 3.33 Mg ha^−1^). Cluster 3 (LYMN) has medium N application rates (mean 63 kg N ha^−1^) and low yields (actual yield = 1.76 Mg ha^−1^). This group gathers 14% of the data points. Cluster 2 (LYLN) and cluster 5 (MYLN) have low N application rates (mean 10 and 9 kg N ha^−1^, respectively). The difference in their actual yields separates them. Cluster 2 has low yields (mean 1.40 Mg ha^−1^) while cluster 5 has medium yields (mean 3.20 Mg ha^−1^). Around 32% and 18% of the data points belong to Clusters 2 and 5, respectively. PFPN of clusters 6 (Very High Yield and Very High N application rate), 1 (High Yield and High N application rate), and 4 (Medium Yield and High N application rate) were in the optimum range (between 30 and 100 kg grain kg^−1^ N). PFPN of clusters 2 (Low Yield and Low N application rate) and 5 (Medium Yield and Low N application rate) were very high (>100 kg grain kg^−1^ N) while the one of cluster 3 (Low Yield and Medium N application rate) was low (Table S9).

**Fig. 7:**
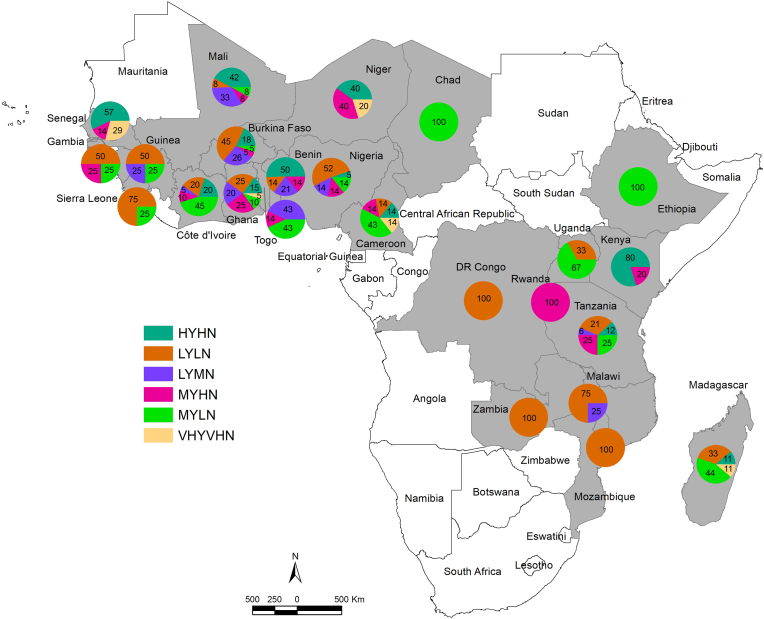
Map displaying pie charts of clusters (combining actual yield and N application rate) for 24 sub-Saharan African countries. HYHN - High Yield and High N application rate; LYLN - Low Yield and Low N application rate; LYMN - Low Yield and Medium N application rate; MYHN -Medium Yield and High N application rate; MYLN - Medium Yield and Low N application rate; VHYVHN - Very High Yield and Very High N application rate.

In CA and ESA, 44% and 27% of data points were in Medium Yield and Low N application while 11% and 16% were in Low Yield and Medium N application and Medium Yield and High N application groups, respectively. Conversely, in WA, 14% of data points were in Medium Yield and Low N application while 31% were in Low Yield and Medium N application and Medium Yield and High N application groups, respectively (Table S10). In irrigated lowland, rainfed lowland, and upland, 17%, 26%, and 17% of data points were in Medium Yield and Low N application while 22%, 34%, and 26 of data points were in Low Yield and Medium N application and Medium Yield and High N application groups, respectively (Table S10). In Côte d’Ivoire, Togo, Cameroon, Chad, Ethiopia, Uganda, and Madagascar, more than 40% of the data points belong to the Medium Yield and Low N application group. In Rwanda, Togo, Ghana, Mali, and Niger, more than 40% of the data points belong to the groups Low Yield and Medium N application and Medium Yield and High N application (Fig. 7 and Table S10).

## 4 Discussion

This study showed a huge spatial variation in fertilizer use in rice fields in SSA. The impact of nutrient application rates on yield and yield gap differed across regions, growing environments, and AEZs. This paper did not intend to provide average fertilizer use in rice fields at continental, sub-regional levels, or for each rice-growing environment. Therefore, the averages shown in this synthesis paper should be taken with caution as they present some biases. Data were not from all the rice-producing countries in SSA, and the averages were not weighted by the cultivation area of each growing environment. This reminds the importance of national programs to monitor fertilizer use for each crop as this information is crucial for planning and decision-making. With that in mind, the synopses of this study have been discussed in three strands: (i) the association between fertilizer use and yield; then, (ii) factors affecting partial factor productivity of nutrients in rice fields; and, (iii) general research and development recommendations for the clusters identified based on actual yield and N application rates.

### 4.1 Importance of fertilizer use in narrowing the yield gap

The huge variability of fertilizer use in the fields reminds the specificity of each region, rice-growing environment, and AEZ, which could be explained by biophysical, economic as well as political factors. For example, N and P fertilizer rates were higher in irrigated lowlands than in rainfed systems. Indeed, rainfed rice farmers give priority to reducing drought and climate variability risks, even in those rainfed lowlands having favorable field water conditions (Tanaka et al., 2017). So, farmers from these systems (rainfed lowland and upland) are generally reluctant to make high investments in fertilizer inputs (Ibrahim et al., 2022; Niang et al., 2017). These biophysical constraints explain the lower fertilizer application rate in rainfed systems (Awio et al., 2021). This emphasizes the importance of water control in rainfed lowland fields in SSA to make this system more stable and productive. We also showed that farmers from the Arid zone applied more fertilizer than those from other AEZs. In the data gathered, all data from the Arid zone were irrigated rice. This conclusion is most likely accurate as irrigated lowland fields are predominant in this zone (Seck et al., 2010). At the regional level, the N, P, and K application rates in WA were higher than those in CA and ESA. Potential reasons could be the higher consumption per capita (i.e., higher demand) (FAO, 2021) and/or fertilizer subsidies (Carter et al., 2016; Tsujimoto et al., 2019). Due to an insufficient number of data points available at the country level, we were unable to test these hypotheses on a broader scale (sub-regional or continental scales).

Higher N and P fertilizer application rates were associated with higher yields and lower yield gaps in irrigated lowlands. This agrees with earlier findings on the positive effects of N and P on yield in irrigated lowlands (Arouna et al., 2021a; Ibrahim et al., 2022). Although specific to irrigated lowlands, this finding supports that increasing the use of fertilizers is critical for improving food security in SSA (Dobermann, 2022). Southeast Asia is a striking example. From 1971 to 2001, the N application rate increased 20-fold leading to a substantial increase in rice yields (IFA/IFDC/IPI/PPI/FAO, 2002). In rainfed lowlands and uplands, water limitations could have hindered the positive effects of the N and P fertilizers (Asai et al., 2021; Niang et al., 2018; Tanaka et al., 2017). Moreover, the rates were most likely too low to allow significant and visible effects.

Although there is growing evidence of K deficiency in rice fields in SSA (Johnson et al., 2021), the K application rate was not correlated with yield and yield gap in any growing environments. Previous studies also demonstrated that yield response to K fertilizer was lower than those of N and P both in rice and maize fields (Zingore et al., 2022). In the Senegal River Valley (Senegal) and Office du Niger (Mali), it is known that considerable amounts of K were provided through irrigation water and dust deposition (Chivenge et al., 2022; Haefele, 2001). Unfortunately, this type of data on N, P, and K inputs is missing in many irrigation schemes in SSA. These observations point out the need for future research addressing site-specific interactions between the applied nutrients and focusing on the optimization of K application rates.

### 4.2 Factors affecting partial factor productivity of nutrients in rice fields

This paper assessed potential risks associated with nutrient surpluses or soil nutrient mining. We found that about 40% of the data points had high partial factor productivity (PFP) of N, P, and K compared to the optimum level, implying an insufficient supply of fertilizer and a high risk of soil nutrient mining (Devkota et al., 2021; Fixen et al., 2015). This characteristic of the nutrient status in rice fields is identical to the whole cropland in SSA. Between 1961 and 1998, depletion rates of N, P, and K in cropland increased dramatically by 225%, 233%, and 256%, respectively (Sheldrick and Lingard, 2004). This is explained by the non-application or suboptimal nutrient application rates throughout the 20th Century (Bouwman et al., 2017). In addition to soil nutrient depletion, this is partly responsible for low crop yields (Vitousek et al., 2009) and in turn, threatens food security. In this study, risks of P and K soil mining were found to be more prevalent (54 and 44%, respectively) than N soil mining (37%). This was not surprising as P availability is one of the major barriers due to its low historical use in agriculture (Magnone et al., 2022). Likewise, this finding confirms a previous study (Majumdar et al., 2021) showing significant negative K balances in SSA. Potassium depletion in soil has been exacerbated by the removal of rice straw for feeding cattle or burning, post-harvesting practices commonly observed in smallholder farmers’ fields in many SSA countries (Johnson et al., 2023; Niang et al., 2017). One estimated that due to the minimal P application rates in the past decades, its application in cropland must increase fivefold from 4 kg ha^−1^ in 2007 to 23 kg ha^−1^ in 2050 to sustain food production in Africa (Sattari et al., 2012). This interpretation of the high risk of soil nutrient mining based on the high partial factor productivity should be taken cautiously, especially in lowland growing environments which have a nutrient balancing system through the nutrition inflows from irrigation water (Dobermann et al., 1998), high N fixation, and slow decomposition of organic carbon (Pampolino et al., 2008). These factors should be included in the calculation of the nutrient balance, thereby enabling a comprehensive assessment of the nutrient depletion in each rice-growing environment.

The variations in partial factor productivity of nutrients were related to the rice-growing environment and AEZ. Irrigated lowlands tended to have higher PFPN than rainfed lowlands and uplands as irrigated lowland systems are more efficiently managed with less water stress leading to higher yield (Dobermann, 2005; Karmakar et al., 2021; Niang et al., 2018). Most rainfed systems are characterized by alternating aerobic and anaerobic soil conditions. This cyclic pattern promotes nitrification in unsaturated soil, which subsequently results in nitrogen loss through denitrification when the soil is saturated. Consequently, this contributes to lower nitrogen-use efficiency in rainfed systems (Buresh, 2015). Another explanation of the outperformance of irrigated lowlands in terms of PFPN could be the impact of soil texture. Ye et al. (2007) demonstrated that soil texture is a key driver of partial factor productivity of N, with clay soil exhibiting higher levels compared to sandy soil. Higher clay content enlarged the soil surface area, allowing for greater adsorption of ammonia ions that move gradually through the soil to the roots of rice plants (Hamoud, 2019). Considering that in SSA, the soils in irrigated lowland rice fields have generally a higher clay content than those in rainfed systems (Johnson et al., 2019; Niang et al., 2017), our finding aligns with the study of Ye et al. (2007). In addition, our research uncovered that a higher P application rate improved PFPN. Indeed, P is a limiting factor in many SSA rice fields (Johnson et al., 2019) because of the high P-fixation in some soils (Sanchez et al., 2003) and the low P input. Under limited P supply, rice cannot achieve its full yield potential. This explains that an increase in P input improves PFPN. Additional factors not considered in this study, such as soil organic matter content and season could also influence the partial factor productivity of applied nutrients. Overall, improving the PFP of nutrients requires an integrated and system-specific management of fertilizers (Awio et al., 2023; Chivenge et al., 2021). An increase in nutrient input is needed to improve yields while reducing soil nutrient depletion.

### 4.3 Clustering and research and development recommendations

Based on actual yield and N application rate, we identified six clusters. The research and development recommendations for each cluster are different (Table 1). Clusters 1 (High Yield and High N application rate) and 6 (Very High Yield and Very High N application rate) perform well and there is little room for improvement. The focus should be on increasing nutrient use efficiency and avoiding N surpluses and losses. Cluster 2 (Low Yield and Low N application rate) has a low socio-economic relevance as rice production was most likely a low priority for some of these areas. About 90% of the data points from this cluster are from rainfed systems, indicating a higher susceptibility to drought and potential mismanagement of water resources. Unraveling the causes of low yields could provide the way forward. A high proportion (>40%) of data points from the Democratic Republic of Congo, Zambia, Mozambique, Malawi, Sierra Leone, The Gambia, Guinea, Nigeria, and Burkina Faso belong to Cluster 2 (Low Yield and Low N application rate). The recommendation for clusters 3 (Low Yield and Medium N application rate) and 4 (Medium Yield and High N application rate) are the same. Both have high socio-economic relevance. In Rwanda, Togo, Ghana, Mali, and Niger, more than 40% of the data points belong to these groups. National and/or international research institutes should investigate the causes of low and medium yields and provide possible solutions. Governments and international agencies should disseminate and promote validated site-specific nutrient management (SSNM) solutions and good agricultural practices (Arouna et al., 2021c; Zossou et al., 2020). The ultimate goal is to improve nutrient use efficiency. There is room for increasing fertilizer inputs in cluster 5 (Medium Yield and Low N application rate). Almost half of this cluster is from irrigated lowlands. A high proportion (>40%) of data points from Ethiopia, Chad, Uganda, Côte d’Ivoire, Togo, and Cameroon belong to this group. Research institutes should investigate reasons for low fertilizer inputs. For irrigated lowland sites, if relevant and according to the country's priorities, governments could establish policies facilitating access to fertilizer for smallholder farmers (Seck et al., 2010). Nigeria and several other countries in SSA (e.g., Senegal, Mali, Burkina Faso, Ghana, Ethiopia, Tanzania, and Malawi) already implement these recommendations and allocate a substantial portion of their agricultural budget for fertilizer subsidies (Jayne and Rashid, 2013; Takeshima and Liverpool-Tasie, 2015). However, in some cases, subsidized fertilizer accounted for a relatively small share of the total fertilizer used in rice fields (Takeshima and Liverpool-Tasie, 2015). Considering that an important share of fertilizers from fertilizer subsidy programs in SSA is diverted before reaching the intended beneficiary farmers, it is crucial to put more emphasis on their design and implementation and to progress toward the new generation of smart subsidy programs (Jayne et al., 2013). For rainfed sites, as fertilizer application is somehow a risky investment because climate variability and water management are more critical issues, governments should be more cautious.

**Table 1:** Research and Development recommendations for the six groups identified by cluster analysis.

Cluster	Research and Development recommendation
Cluster 1: High Yield and High N application rate (HYHN)	Little room for yield improvement. Focus on increasing nutrient use efficiency.

Cluster 2: Low Yield and Low N application rate (LYLN)	Low socio-economic relevance i.e., Research and Development actions have a limited impact on farmers' livelihoods and the economic development of the community. Unraveling the causes of low yields could provide the way forward.

Cluster 3: Low Yield and Medium N application rate (LYMN)	High socio-economic relevance i.e., Research and Development actions could have substantial implications for improving farmers' well-being, addressing socio-economic inequalities, and contributing to economic growth in the area. Research institutes should investigate the yield gap, causes of low yield, and possible solutions. Site-specific nutrient management (SSNM) solutions should be tested. Then, governments/agencies should disseminate and promote good agricultural practices and SSNM solutions.

Cluster 4: Medium Yield and High N application rate (MYHN)	High socio-economic relevance. Research institutes should investigate the yield gap, causes of medium yield, and possible solutions. Site-specific nutrient management (SSNM) solutions should be tested. Then, governments/agencies should disseminate and promote good agricultural practices and SSNM solutions.

Cluster 5: Medium Yield and Low N application rate (MYLN)	Potential for further increasing fertilizer inputs; Research institutes should investigate reasons for low fertilizer inputs. Then, if relevant and according to the country's priorities, governments could establish policies facilitating access to fertilizer for smallholder farmers.

Cluster 6: Very High Yield and Very High N application rate (VHYVHN)	Little room for yield improvement. Focus on increasing input use efficiency.

Although the research and development recommendations provided here are quite general, we identified priority countries or sites where more research and development resources should be invested using limited data. However, this simple analysis presents some weaknesses and limitations that could be improved in future studies. First, as mentioned earlier, few data points are available per country and site, making general conclusions at national or sub-national levels less accurate. Second, a classification based on yield gaps and N inputs would have been more adequate to draw more relevant research and development recommendations. We did not include yield gap data in our classification as potential yield or water-limited potential yield was not available for more than half of the data points. Further efforts in modeling yield potential and water-limited yield potential for the new sites are needed to fill this gap. In addition, as financial issues, risks for water stress, and climate variability could be key reasons explaining low fertilizer inputs in SSA, further studies including fertilizer prices and other socio-economic and climatic parameters could help to fine-tune these recommendations.

## 5 Conclusion

By reviewing the fertilizer use, grain yield, and relative yield gap in smallholder farmer rice fields in 24 countries in sub-Saharan Africa over the last three decades, this study highlighted, for the first time, a large variation of N, P, and K application rates across regions, agroecological zones, and growing environments. The N, P, and K application rates were higher in West Africa than in Central and East, and Southern Africa, and in irrigated lowlands than in rainfed systems. An insufficient supply of fertilizer implying a high risk of soil nutrient mining was reported in almost half of the cases. N and P application rates had a strong effect on the reduction of the relative yield gap in irrigated lowlands while no effect was found in rainfed systems. Reasons for low fertilizer use and its poor association with rice yield need also to be identified for developing a strategy for enhancing its use and reducing the yield gaps. Moreover, as the records of fertilizer application rates in farmers' fields in sub-Saharan Africa are scarce and data obtained in this review is limited, we, therefore, recommend the development of nutrient use efficiency monitoring systems in sub-Saharan Africa countries for long-term trend analysis, nutrient balance assessment, and fertilizer demand forecasts. Such strategies would help increase rice yield, reduce rice import dependency in many sub-Saharan African countries, sustain soil fertility, and, by implication, improve global food security.

## Funding

The authors gratefully acknowledge the 10.13039/100000865Bill & Melinda Gates Foundation (BMGF, Seattle, USA; Grant ID INV-005431) for supporting this study through the 10.13039/501100015815CGIAR Excellence in Agronomy (2030) project (Incubation Phase).

## Authors’ contributions

**J.-M.J.**: Conceptualization, Methodology, Data collection, curation & analysis, Visualization, Writing - Original Draft; **I.A.**: Data collection, Writing - Review & Editing; **E.R.D.-Y.**: Data collection, Writing - Review & Editing; **K·S.**: Data collection, Writing - Review & Editing; **Y.T.**: Data collection, Writing - Review & Editing; **H.A.**: Data collection, Writing - Review & Editing; **KS**: Conceptualization, Methodology, Data collection, Writing - Review & Editing.

## Declaration of competing interest

The authors declare that they have no known competing financial interests or personal relationships that could have appeared to influence the work reported in this paper.

## Data Availability

Data will be made available on request.
