# Supplementary material for: Inorganic fertilizer use and its association with rice yield gaps in sub-Saharan Africa
Source: Glob Food Sec. 2023 Sep;38:100708. doi: 10.1016/j.gfs.2023.100708 (PMC10518462; doi:10.1016/j.gfs.2023.100708)
Supplement: Multimedia component 1 [file mmc1.pdf]

Supplementary material

Global Food Security

Inorganic fertilizer use and its association with rice yield gaps in sub-Saharan Africa

Jean-Martial Johnson[1, 2*], Ali Ibrahim[3], Elliott Ronald Dossou-Yovo[1], Kalimuthu Senthilkumar[4], Yasuhiro Tsujimoto[5], Hidetoshi Asai[5], Kazuki Saito[1]

[1] Africa Rice Center (AfricaRice), 01 B.P. 2551, Bouaké 01, Côte d'Ivoire

[2] Institute of Crop Science and Resource Conservation (INRES), University of Bonn, D-53115 Bonn, Germany

[3] Africa Rice Center (AfricaRice), PMB 82, 901101, Abuja, Nigeria

[4] Africa Rice Center (AfricaRice), B.P. 1690, Antananarivo 101, Madagascar

[5] Japan International Research Center for Agricultural Sciences (JIRCAS), 1-1 Ohwashi, Tsukuba, Ibaraki 3058686, Japan

* Corresponding author: Jean-Martial Johnson, E-mail address: j.johnson@uni-bonn.de; https://orcid.org/0000-0002-2638-8774

Table S1

Mean grain yield and fertilizer application rates across various countries and rice production systems in sub-Saharan Africa.

Country	Growing environment	Grain yield (Mg/ha)				N application rate (kg N/ha)				P application rate (kg P/ha)				K application rate (kg K/ha)			
		n	Mean	SD	Median	n	Mean	SD	Median	n	Mean	SD	Median	n	Mean	SD	Median
Benin	IL	9	4.57	0.94	3.20	9	92	17	67	9	16	4	10	9	29	8	18
Benin	RL	4	1.80	0.71	0.98	4	41	38	7	4	6	5	3	4	11	9	5
Burkina Faso	IL	8	5.37	0.65	4.38	8	95	20	77	8	19	3	15	8	20	0	20
Burkina Faso	RL	2	2.64	0.15	2.54	2	46	50	14	2	12	8	7	2	7		7
Cameroon	IL	2	2.85	0.46	2.55	2	29	31	9	2	6	7	2	2	9	8	4
Cameroon	RL	5	3.83	1.66	1.75	5	43	70	0	5	2	2	0	5	4	5	0
Côte d'Ivoire	IL	10	3.93	1.20	2.24	10	63	37	11	10	20	16	5	10	27	14	8
Côte d'Ivoire	RL	2	2.41	1.48	1.47	2	23	11	16	2	3	2	2	2	5	3	3
Côte d'Ivoire	RU	3	1.74	0.79	1.16	3	0	0	0	3	0	0	0	3	0	0	0
Ghana	IL	8	4.51	0.78	3.54	8	95	45	34	8	17	9	5	8	21	12	7
Ghana	RL	9	2.29	2.07	0.86	9	49	45	6	9	9	10	1	9	10	10	2
Ghana	RU	3	1.96	0.53	1.50	3	33	21	16	3	4	2	3	3	5		5
Guinea	RU	2	1.54	0.18	1.42	2	8	5	4	2	2	0	2	2	4	0	3
Kenya	IL	3	5.02	0.30	4.75	3	53	16	40	3	25	2	24	3	3		3
Madagascar	RL	2	3.61	0.47	3.31	2	0	0	0	2	0		0	2	0		0
Madagascar	RU	3	2.15	0.53	1.82	3	5	7	0	3	0	0	0	3	0	0	0
Malawi	RL	2	1.70	0.55	1.35	2	11		11	2	2		2	2	0		0
Malawi	RU	2	1.14	0.57	0.78	2	18		18	2	4		4	2	0		0
Mali	IL	6	4.82	1.15	3.12	6	86	43	28	6	15	9	4	6	3	2	1
Mali	RL	3	1.95	0.59	1.45	3	55	28	30	3	6	4	3	3	7	4	4
Mali	RU	2	1.28	0.81	0.77	2	66	32	46	2	12	4	9	2	8	12	1
Niger	IL	4	4.79	1.40	3.12	4	113	41	85	4	15	7	7	4	25	13	13
Nigeria	IL	4	4.53	0.52	3.94	4	50	52	4	4	9	8	1	4	0		0
Nigeria	RL	8	2.24	0.81	1.23	8	40	34	2	8	6	6	0	8	0	0	0
Nigeria	RU	2	1.67	0.23	1.53	2	33	26	17	2	9		9	2			

Senegal	IL	17	5.30	1.02	3.54	17	144	52	78	17	25	16	8	17	1	2	0
Sierra Leone	RL	4	1.66	0.88	0.89	4	8	13	0	4	1	2	0	4	3	3	0
Tanzania	IL	5	4.07	1.74	2.40	5	56	41	10	5	12	8	3	5	0		0
Tanzania	RL	6	3.38	1.46	2.07	6	38	35	5	6	3	3	0	6	0	0	0
The Gambia	IL	2	3.67	0.11	3.60	2	44	35	22	2	7	5	4	2	13	9	7
The Gambia	RU	2	1.32	0.30	1.12	2	13	4	10	2	4	1	3	2	7	3	6
Togo	IL	3	3.65	0.99	2.67	3	23	20	3	3	6	6	0	3	10	12	1
Togo	RL	3	2.58	0.61	2.02	3	56	73	3	3	9	8	1	3	16	14	2
Uganda	IL	2	3.03	0.11	2.96	2	1	1	0	2	0		0	2	0		0

n - Number of data points

SD - Standard error

IL – Irrigated lowland

RL – Rainfed lowland

RU – Rainfed upland

Table S2

Comparison of N, P, K application rates, partial factor productivity of N, P, and K and grain yield, and relative yield gap in rice fields between coastal and landlocked countries in sub-Saharan Africa.

	Coastal country	Landlocked country	Test statistic
N application rate (kg N/ha)			0.475
Mean (SD)	48.95 (50.72)	50.15 (44.07)	Wilcoxon rank-sum
Median (IQR)	30.00 (71.10)	44.55 (69.62)	
P application rate (kg P/ha)			0.369
Mean (SD)	9.26 (11.01)	9.24 (8.56)	Wilcoxon rank-sum
Median (IQR)	5.20 (12.50)	6.30 (15.57)	
K application rate (kg K/ha)			0.105
Mean (SD)	8.31 (11.66)	8.68 (9.34)	Wilcoxon rank-sum
Median (IQR)	3.05 (11.15)	4.30 (15.60)	
Grain yield (Mg grain/ha)			0.008
Mean (SD)	3.14 (1.64)	2.60 (1.70)	Wilcoxon rank-sum
Median (IQR)	2.76 (2.60)	1.95 (2.05)	
PFPN (kg grain/kg N)			0.015
Mean (SD)	195.46 (343.18)	177.83 (374.53)	Wilcoxon rank-sum
Median (IQR)	71.00 (144.50)	53.00 (85.00)	
PFPP (kg grain/kg P)			0.014
Mean (SD)	1774.28 (8528.12)	483.34 (619.90)	Wilcoxon rank-sum
Median (IQR)	367.50 (827.50)	281.00 (284.25)	
PFPK (kg grain/kg K)			0.018
Mean (SD)	940.00 (2198.31)	359.70 (383.41)	Wilcoxon rank-sum
Median (IQR)	306.00 (642.00)	171.50 (380.25)	
Potential yield or water-limited yield (Mg/ha)			0.025
Mean (SD)	7.85 (1.64)	8.66 (1.62)	Wilcoxon rank-sum
Median (IQR)	7.60 (2.00)	8.70 (1.00)	
Relative yield gap (%)			0.344
Mean (SD)	57.03 (22.30)	53.77 (18.29)	Wilcoxon rank-sum
Median (IQR)	61.40 (31.60)	46.90 (28.60)	

SD - Standard deviation

IQR - Interquartile range

Table S3

N, P, K application rates, partial factor productivity of N, P and K, grain yield, and relative yield gap in rice fields, across various regions in sub-Saharan Africa.

	WA	CA	ESA	Overall	Test statistic
N application rate (kg N/ha)					<0.001
Mean (SD)	57.91 (50.21)	30.77 (53.74)	24.73 (31.51)	49.31 (48.74)	Kruskal-Wallis
Median (IQR)	52.10 (73.83)	7.20 (43.00)	10.00 (39.80)	30.30 (72.00)	
Group	a	b	b		
P application rate (kg P/ha)					<0.001
Mean (SD)	10.77 (10.77)	2.63 (3.69)	5.16 (7.37)	9.25 (10.27)	Kruskal-Wallis
Median (IQR)	9.00 (14.40)	1.60 (4.00)	1.60 (7.20)	5.35 (13.33)	
Group	a	b	b		
K application rate (kg K/ha)					<0.001
Mean (SD)	10.66 (11.58)	4.22 (5.20)	1.19 (3.75)	8.43 (10.93)	Kruskal-Wallis
Median (IQR)	5.30 (16.88)	3.00 (7.60)	0.00 (1.03)	3.60 (16.00)	
Group	a	ab	b		
PFPN (kg grain/ha/kg N)					<0.001
Mean (SD)	134.33 (245.56)	401.67 (649.17)	365.76 (535.13)	189.84 (352.64)	Kruskal-Wallis
Median (IQR)	54.00 (86.00)	128.00 (227.00)	157.00 (229.25)	63.00 (133.50)	
Group	b	ab	a		
PFPP (kg grain/kg P)					0.005
Mean (SD)	1374 (7748)	995 (476)	1157 (1248)	1325 (6911)	Kruskal-Wallis
Median (IQR)	299 (345)	1062 (251)	609 (1358)	325 (635)	
Group	a	ab	b		
PFPK (kg grain/kg K)					0.001
Mean (SD)	666 (1830)	536.60 (223.77)	1154.64 (869.92)	704.11 (1730.02)	Kruskal-Wallis
Median (IQR)	203 (395)	558 (141)	783 (1077)	249 (552)	
Group	a	ab	a		

Grain yield (Mg grain/ha)					0.638
Mean (SD)	3.03 (1.70)	3.15 (1.53)	2.80 (1.65)	2.98 (1.68)	Kruskal-Wallis
Median (IQR)	2.55 (2.98)	2.85 (2.03)	2.30 (1.99)	2.55 (2.77)	
Group	a	a	a		
Potential yield or water-limited yield (Mg/ha)					0.163
Mean (SD)	7.87 (1.33)	8.44 (1.26)	9.56 (2.92)	8.10 (1.67)	Kruskal-Wallis
Median (IQR)	8.10 (1.95)	8.90 (0.20)	9.80 (4.52)	8.15 (2.20)	
Group	a	a	a		
Relative yield gap (%)					0.727
Mean (SD)	55.36 (21.82)	56.42 (13.31)	60.43 (19.22)	56.02 (21.10)	Kruskal-Wallis
Median (IQR)	56.50 (35.30)	58.70 (14.40)	66.25 (13.28)	60.05 (32.25)	
Group	a	a	a		

WA - West Africa

CA - Central Africa

ESA - East and Southern Africa

Table S4

N, P, K application rates, partial factor productivity of N, P, and K and grain yield in rice fields, and relative yield gap across various growing environments [irrigated lowland (IL), rainfed lowland (RL), and, rainfed upland (RU)] in sub-Saharan Africa.

	IL	RL	RU	Test statistic
N application rate (kg N/ha)				<0.001
Mean (SD)	84.17 (70.32)	38.46 (43.05)	23.25 (28.41)	Kruskal-Wallis
Median (IQR)	86.90 (62.50)	25.30 (58.20)	12.60 (34.25)	
Group	a	b	b	
P application rate (kg P/ha)				<0.001
Mean (SD)	16.78 (11.54)	5.56 (6.43)	4.16 (4.38)	Kruskal-Wallis
Median (IQR)	15.95 (10.70)	3.50 (9.55)	2.90 (6.35)	
Group	a	b	b	
K application rate (kg K/ha)				0.004
Mean (SD)	14.87 (13.92)	6.52 (8.86)	4.37 (6.45)	Kruskal-Wallis
Median (IQR)	14.00 (22.95)	4.10 (7.18)	0.00 (5.50)	
Group	a	b	b	
PFPN (kg grain/kg N)				0.57
Mean (SD)	122.45 (259.57)	257.71 (515.29)	205.50 (325.22)	Kruskal-Wallis
Median (IQR)	54.00 (33.00)	74.00 (114.00)	98.00 (168.50)	
Group	a	a	a	
PFPP (kg grain/kg P)				0.419
Mean (SD)	1797.39 (11113.39)	1173.03 (2129.31)	444.00 (367.15)	Kruskal-Wallis
Median (IQR)	299.00 (155.50)	444.00 (671.00)	236.00 (448.50)	
Group	a	a	a	
PFPK (kg grain/kg K)				0.708
Mean (SD)	826.45 (2679.79)	674.84 (1229.89)	269.75 (183.35)	Kruskal-Wallis
Median (IQR)	225.50 (442.50)	327.00 (323.00)	270.00 (292.25)	

Group	a	a	a	
Grain yield (Mg grain/ha)				<0.001
Mean (SD)	4.56 (1.26)	2.54 (1.34)	1.69 (0.57)	Kruskal-Wallis
Median (IQR)	4.70 (1.67)	2.47 (1.66)	1.58 (0.45)	
Group	a	b	c	
Potential yield or water-limited yield (Mg/ha)				<0.001
Mean (SD)	8.70 (1.36)	7.61 (1.85)	6.78 (1.27)	Kruskal-Wallis
Median (IQR)	8.80 (1.90)	7.90 (2.65)	7.10 (0.50)	
Group	a	b	b	
Relative yield gap (%)				
Mean (SD)	45.74 (17.90)	66.35 (19.19)	74.08 (12.58)	<0.001
Median (IQR)	45.00 (25.65)	69.25 (24.17)	76.80 (17.20)	1-way ANOVA
Group	b	a	a	

Table S5

N, P, K application rates, partial factor productivity of N, P and K and grain yield, and relative yield gap in rice fields across various agroecological zones (AEZ) in sub-Saharan Africa.

	Humid	Sub-humid	Semi-Arid	Arid	Highlands	Test statistic
N application rate (kg N/ha)						<0.001
Mean (SD)	32.46 (38.36)	48.95 (43.18)	59.52 (45.49)	139.38 (51.73)	35.53 (47.24)	Kruskal-Wallis
Median (IQR)	14.35 (68.52)	38.10 (73.15)	63.45 (71.00)	136.00 (46.00)	24.65 (45.20)	
Group	b	b	b	a	b	
P application rate (kg P/ha)						0.005
Mean (SD)	5.82 (6.42)	9.15 (9.73)	9.72 (7.59)	18.06 (10.23)	6.95 (10.01)	Kruskal-Wallis
Median (IQR)	2.50 (10.43)	6.00 (12.90)	9.45 (15.02)	18.50 (8.15)	2.40 (8.33)	
Group	b	b	ab	a	b	
K application rate (kg K/ha)						0.025
Mean (SD)	10.98 (12.17)	12.29 (13.42)	9.46 (9.97)	1.21 (2.12)	4.70 (7.43)	Kruskal-Wallis
Median (IQR)	4.25 (20.30)	6.00 (21.90)	5.30 (13.60)	0.00 (1.75)	0.00 (7.60)	
Group	ab	a	ab	b	ab	
PFPN (kg grain/kg N)						0.002
Mean (SD)	327.27 (516.74)	167.27 (369.99)	134.00 (330.23)	42.69 (19.93)	327.00 (459.51)	Kruskal-Wallis
Median (IQR)	86.00 (256.00)	61.00 (104.00)	49.00 (30.25)	39.00 (10.00)	108.00 (144.00)	
Group	ab	ab	bc	c	a	
PFPP (kg grain/kg P)						0.626
Mean (SD)	1216.00 (2214.66)	744.13 (1474.51)	2379.78 (13565.04)	472.27 (550.14)	1177.83 (1488.36)	Kruskal-Wallis
Median (IQR)	444.00 (575.00)	302.00 (401.25)	284.00 (200.00)	299.00 (150.50)	717.00 (856.50)	
Group	a	a	a	a	a	
PFPK (kg grain/kg K)						0.131
Mean (SD)	510.44 (740.91)	460.05 (934.67)	833.44 (2973.96)	1191.50 (125.16)	745.75 (645.99)	Kruskal-Wallis
Median (IQR)	234.00 (295.00)	178.00 (317.25)	222.00 (171.00)	1191.50 (88.50)	578.00 (407.25)	
Group	a	a	a	a	a	

Grain yield (Mg grain/ha)						<0.001
Mean (SD)	2.51 (1.23)	3.10 (1.58)	3.12 (1.86)	5.30 (1.02)	3.88 (1.28)	Kruskal-Wallis
Median (IQR)	2.44 (2.11)	2.70 (2.24)	2.76 (3.51)	5.56 (1.10)	3.91 (1.56)	
Group	b	b	b	a	ab	
Potential yield or water-limited yield (Mg/ha)						<0.001
Mean (SD)	9.39 (1.61)	7.33 (1.74)	8.35 (1.11)	7.23 (0.51)	10.32 (2.00)	Kruskal-Wallis
Median (IQR)	9.40 (1.08)	7.10 (2.75)	8.60 (1.15)	7.40 (0.00)	9.40 (1.10)	
Group	a	c	ab	bc	a	
Relative yield gap (%)						
Mean (SD)	73.63 (12.32)	60.96 (20.30)	53.62 (17.74)	27.18 (11.91)	57.66 (13.86)	<0.001
Median (IQR)	76.50 (19.60)	65.80 (26.85)	48.20 (25.05)	25.80 (16.50)	63.30 (16.30)	1-way ANOVA
Group	a	ab	b	c	ab	

Table S6

Proportion (%) of studies having optimum levels of partial factor productivity of N (PFPN), P (PFPP), and K (PFPK) across various regions [West Africa (WA), Central Africa (CA), and East and Southern Africa (ESA)] in sub-Saharan Africa

Indicators threshold levels	WA	CA	ESA	Overall
Partial factor productivity of N (PFPN) (kg grain kg^{-1} N)				
Very low (wasteful application)	17.9	0.0	4.8	14.7
Optimum range	52.6	33.3	33.3	48.0
Very high (risk of soil mining)	28.8	66.7	61.9	36.8
Partial factor productivity of P (PFPP) (kg grain kg^{-1} P)				
Very low (wasteful application)	5.7	0.0	0.0	4.5
Optimum range	44.7	20.0	28.1	41.0
Very high (risk of soil mining)	49.6	80.0	71.9	54.5
Partial factor productivity of K (PFPK) (kg grain kg^{-1} K)				
Very low (wasteful application)	1.9	0.0	0.0	1.6
Optimum range	60.7	20.0	9.1	54.5
Very high (risk of soil mining)	37.4	80.0	90.9	43.9

For PFPN, Very low (wasteful application, < 30); Optimum range (30 - 100); Very high (risk of soil mining, > 100)

For PFPP, Very low (wasteful application, < 100); Optimum range (100 - 300); Very high (risk of soil mining, > 300)

For PFPK, Very low (wasteful application, < 50); Optimum range (50 - 300); Very high (risk of soil mining, > 300)

Table S7

Proportion (%) of studies having optimum levels of partial factor productivity of N (PFPN), P (PFPP), and K (PFPK) across various growing environments [irrigated lowland (IL), rainfed lowland (RL) and, rainfed upland (RU)] in sub-Saharan Africa

Indicators threshold levels	IL	RL	RU	Overall
Partial factor productivity of N (PFPN) (kg grain kg^{-1} N)				
Very low (wasteful application)	4.9	15.6	22.2	14.7
Optimum range	74.1	42.2	27.8	48.0
Very high (risk of soil mining)	21.0	40.0	50.0	36.8
Partial factor productivity of P (PFPP) (kg grain kg^{-1} P)				
Very low (wasteful application)	0.0	0.0	7.1	4.5
Optimum range	50.0	43.2	50.0	41.0
Very high (risk of soil mining)	50.0	56.8	42.9	54.5
Partial factor productivity of K (PFPK) (kg grain kg^{-1} K)				
Very low (wasteful application)	0.0	0.0	12.5	1.6
Optimum range	66.7	48.0	50.0	54.5
Very high (risk of soil mining)	33.3	52.0	37.5	43.9

For PFPN, Very low (wasteful application, < 30); Optimum range (30 - 100); Very high (risk of soil mining, > 100)

For PFPP, Very low (wasteful application, < 100); Optimum range (100 - 300); Very high (risk of soil mining, > 300)

For PFPK, Very low (wasteful application, < 50); Optimum range (50 - 300); Very high (risk of soil mining, > 300)

Table S8

Proportion (%) of data points having optimum levels of partial factor productivity of N (PFPN), P (PFPP), and K (PFPK) across various agroecological zones (AEZ) in sub-Saharan Africa

Indicators threshold levels	Humid	Sub-Humid	Semi-Arid	Arid	Highlands	Overall
Partial factor productivity of N (PFPN) (kg grain kg^{-1} N)						
Very low (wasteful application)	9.1	13.4	12.5	15.4	0.0	14.7
Optimum range	54.5	52.2	70.8	76.9	33.3	48.0
Very high (risk of soil mining)	36.4	32.8	16.7	7.7	66.7	36.8
Partial factor productivity of P (PFPP) (kg grain kg^{-1} P)						
Very low (wasteful application)	0.0	0.0	4.4	0.0	0.0	4.5
Optimum range	33.3	50.0	48.9	54.5	33.3	41.0
Very high (risk of soil mining)	66.7	50.0	46.7	45.5	66.7	54.5
Partial factor productivity of K (PFPK) (kg grain kg^{-1} K)						
Very low (wasteful application)	0.0	0.0	2.9	0.0	0.0	1.6
Optimum range	55.6	64.3	70.6	0.0	25.0	54.5
Very high (risk of soil mining)	44.4	35.7	26.5	100.0	75.0	43.9

For PFPN, Very low (wasteful application, < 30); Optimum range (30 - 100); Very high (risk of soil mining, > 100)

For PFPP, Very low (wasteful application, < 100); Optimum range (100 - 300); Very high (risk of soil mining, > 300)

For PFPK, Very low (wasteful application, < 50); Optimum range (50 - 300); Very high (risk of soil mining, > 300)

Table S9

Average values of fertilizer inputs and grain yield across groups identified by cluster analysis

	HYHN	LYLN	LYMN	MYHN	MYLN	VHYVHN
N application rate (kg N/ha)						
Count	45	71	31	28	41	8
Mean (SD)	93 (27)	10 (8)	63 (17)	87 (32)	9 (9)	171 (46)
Median (IQR)	96 (36)	8 (12)	63 (28)	78 (24)	6 (14)	164 (17)
P application rate (kg P/ha)						
Mean (SD)	18 (8)	2 (1)	13 (7)	13 (6)	2 (3)	23 (12)
Median (IQR)	18 (6)	1 (2)	10 (6)	12 (6)	1 (2)	22 (10)
K application rate (kg K/ha)						
Mean (SD)	18 (15)	2 (3)	16 (9)	19 (11)	3 (5)	13 (20)
Median (IQR)	18 (27)	1 (4)	18 (4)	21 (15)	0 (4)	5 (18)
Grain yield (Mg grain/ha)						
Mean (SD)	5.16 (0.58)	1.44 (0.51)	1.76 (0.50)	3.33 (0.63)	3.20 (0.69)	6.69 (1.06)
Median (IQR)	5.13 (0.76)	1.48 (0.81)	1.55 (0.64)	3.44 (1.03)	3.12 (1.03)	6.40 (0.64)
PFPN (kg grain/kg N)						
Mean (SD)	62 (25)	275 (379)	32 (19)	43 (17)	544 (585)	42 (16)
Median (IQR)	53 (26)	150 (241)	23 (18)	40 (26)	294 (587)	38 (8)

Cluster 1: High Yield and High N application rate (HYHN); Cluster 2: Low Yield and Low N application rate (LYLN);

Cluster 3: Low Yield and Medium N application rate (LYMN); Cluster 4: Medium Yield and High N application rate (MYHN);

Cluster 5: Medium Yield and Low N application rate (MYLN); Cluster 6: Very High Yield and Very High N application rate (VHYVHN)

Table S10:

Proportions of yield and N input clusters for different regions, rice-growing environments, and countries

	HYHN	LYLN	LYMN	MYHN	MYLN	VHYVHN
**Region**						
Count (%)	45 (20%)	71 (32%)	31 (14%)	28 (12%)	41 (18%)	8 (4%)
(Row %)(Col %)						
WA	37 (23%) (82%)	47 (29%) (66%)	29 (18%) (94%)	21 (13%) (75%)	23 (14%) (56%)	6 (4%) (75%)
CA	1 (11%) (2%)	2 (22%) (3%)	0 (0%) (0%)	1 (11%) (4%)	4 (44%) (10%)	1 (11%) (12%)
ESA	7 (13%) (16%)	22 (42%) (31%)	2 (4%) (6%)	6 (12%) (21%)	14 (27%) (34%)	1 (2%) (12%)
**Growing environment**						
IL	42 (51%) (98%)	2 (2%) (6%)	2 (2%) (12%)	16 (20%) (64%)	14 (17%) (44%)	6 (7%) (75%)
RL	1 (2%) (2%)	18 (34%) (55%)	10 (19%) (59%)	8 (15%) (32%)	14 (26%) (44%)	2 (4%) (25%)
RU	0 (0%) (0%)	13 (57%) (39%)	5 (22%) (29%)	1 (4%) (4%)	4 (17%) (12%)	0 (0%) (0%)
**Country**						
Benin	7 (50%) (16%)	2 (14%) (3%)	3 (21%) (10%)	2 (14%) (7%)	0 (0%) (0%)	0 (0%) (0%)
Burkina Faso	7 (18%) (16%)	17 (45%) (24%)	10 (26%) (32%)	2 (5%) (7%)	2 (5%) (5%)	0 (0%) (0%)
Cameroon	1 (14%) (2%)	1 (14%) (1%)	0 (0%) (0%)	1 (14%) (4%)	3 (43%) (7%)	1 (14%) (12%)
Chad	0 (0%) (0%)	0 (0%) (0%)	0 (0%) (0%)	0 (0%) (0%)	1 (100%) (2%)	0 (0%) (0%)
Côte d'Ivoire	4 (20%) (9%)	4 (20%) (6%)	1 (5%) (3%)	2 (10%) (7%)	9 (45%) (22%)	0 (0%) (0%)
DRC	0 (0%) (0%)	1 (100%) (1%)	0 (0%) (0%)	0 (0%) (0%)	0 (0%) (0%)	0 (0%) (0%)
Ethiopia	0 (0%) (0%)	0 (0%) (0%)	0 (0%) (0%)	0 (0%) (0%)	2 (100%) (5%)	0 (0%) (0%)
Ghana	3 (15%) (7%)	5 (25%) (7%)	4 (20%) (13%)	5 (25%) (18%)	2 (10%) (5%)	1 (5%) (12%)
Guinea	0 (0%) (0%)	2 (50%) (3%)	1 (25%) (3%)	0 (0%) (0%)	1 (25%) (2%)	0 (0%) (0%)
Kenya	4 (80%) (9%)	0 (0%) (0%)	0 (0%) (0%)	1 (20%) (4%)	0 (0%) (0%)	0 (0%) (0%)
Madagascar	1 (11%) (2%)	3 (33%) (4%)	0 (0%) (0%)	0 (0%) (0%)	4 (44%) (10%)	1 (11%) (12%)
Malawi	0 (0%) (0%)	3 (75%) (4%)	1 (25%) (3%)	0 (0%) (0%)	0 (0%) (0%)	0 (0%) (0%)
Mali	5 (42%) (11%)	1 (8%) (1%)	4 (33%) (13%)	1 (8%) (4%)	1 (8%) (2%)	0 (0%) (0%)
Mozambique	0 (0%) (0%)	4 (100%) (6%)	0 (0%) (0%)	0 (0%) (0%)	0 (0%) (0%)	0 (0%) (0%)
Niger	2 (40%) (4%)	0 (0%) (0%)	0 (0%) (0%)	2 (40%) (7%)	0 (0%) (0%)	1 (20%) (12%)
Nigeria	1 (5%) (2%)	11 (52%) (15%)	3 (14%) (10%)	3 (14%) (11%)	3 (14%) (7%)	0 (0%) (0%)
Rwanda	0 (0%) (0%)	0 (0%) (0%)	0 (0%) (0%)	1 (100%) (4%)	0 (0%) (0%)	0 (0%) (0%)
Senegal	8 (57%) (18%)	0 (0%) (0%)	0 (0%) (0%)	2 (14%) (7%)	0 (0%) (0%)	4 (29%) (50%)
Sierra Leone	0 (0%) (0%)	3 (75%) (4%)	0 (0%) (0%)	0 (0%) (0%)	1 (25%) (2%)	0 (0%) (0%)
Tanzania	2 (12%) (4%)	5 (31%) (7%)	1 (6%) (3%)	4 (25%) (14%)	4 (25%) (10%)	0 (0%) (0%)
The Gambia	0 (0%) (0%)	2 (50%) (3%)	0 (0%) (0%)	1 (25%) (4%)	1 (25%) (2%)	0 (0%) (0%)
Togo	0 (0%) (0%)	0 (0%) (0%)	3 (43%) (10%)	1 (14%) (4%)	3 (43%) (7%)	0 (0%) (0%)
Uganda	0 (0%) (0%)	2 (33%) (3%)	0 (0%) (0%)	0 (0%) (0%)	4 (67%) (10%)	0 (0%) (0%)
Zambia	0 (0%) (0%)	5 (100%) (7%)	0 (0%) (0%)	0 (0%) (0%)	0 (0%) (0%)	0 (0%) (0%)

Cluster 1: High Yield and High N application rate (HYHN); Cluster 2: Low Yield and Low N application rate (LYLN);

Cluster 3: Low Yield and Medium N application rate (LYMN); Cluster 4: Medium Yield and High N application rate (MYHN);

Cluster 5: Medium Yield and Low N application rate (MYLN); Cluster 6: Very High Yield and Very High N application rate (VHYVHN)

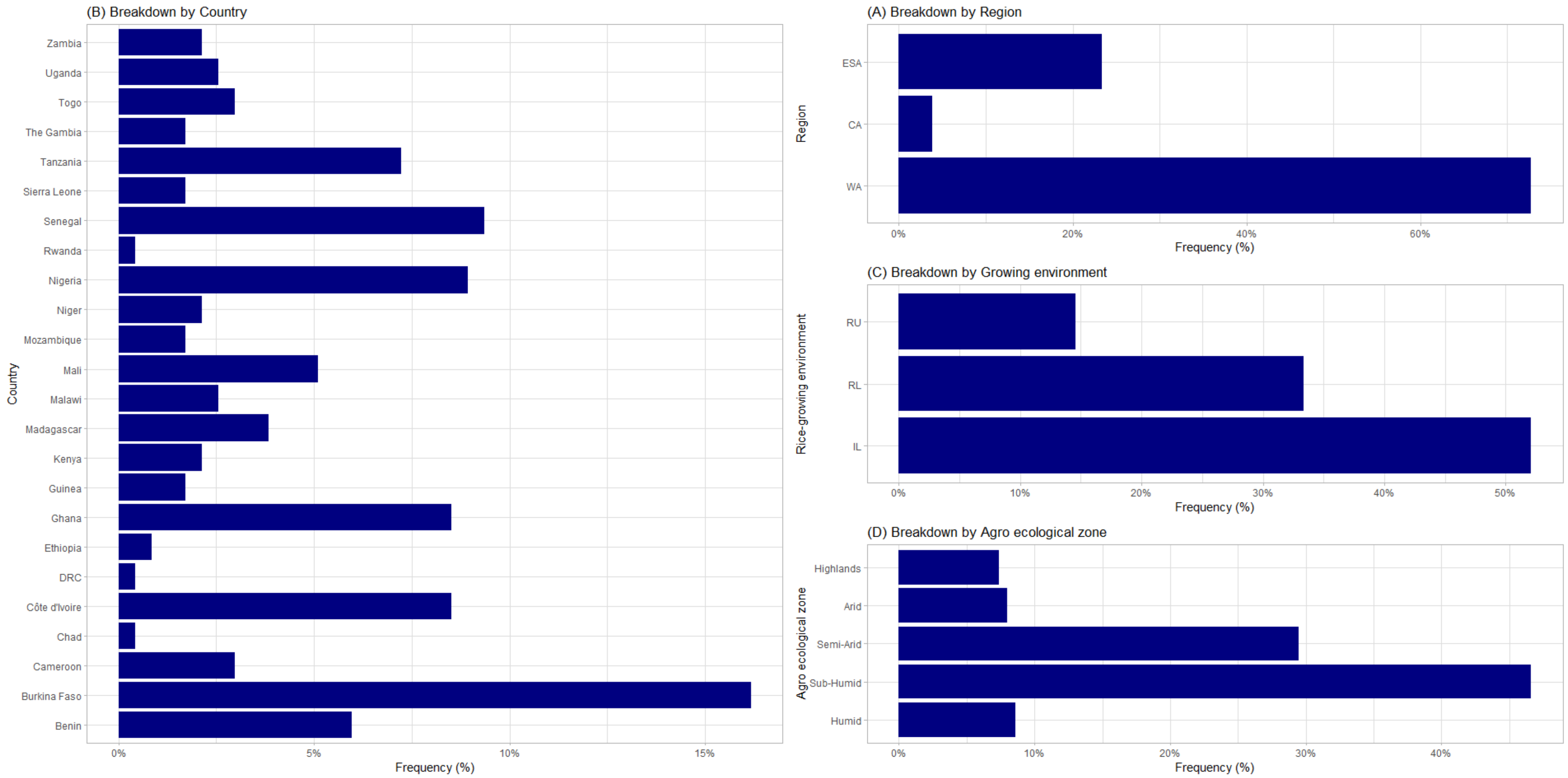

Fig. S1. Data points of fertilizer application rates breakdown by (A) Region; (B) Country; (C) Growing environment and (D) Agroecological zone in rice fields reviewed from studies conducted between 1995 and 2020 in sub-Saharan Africa.

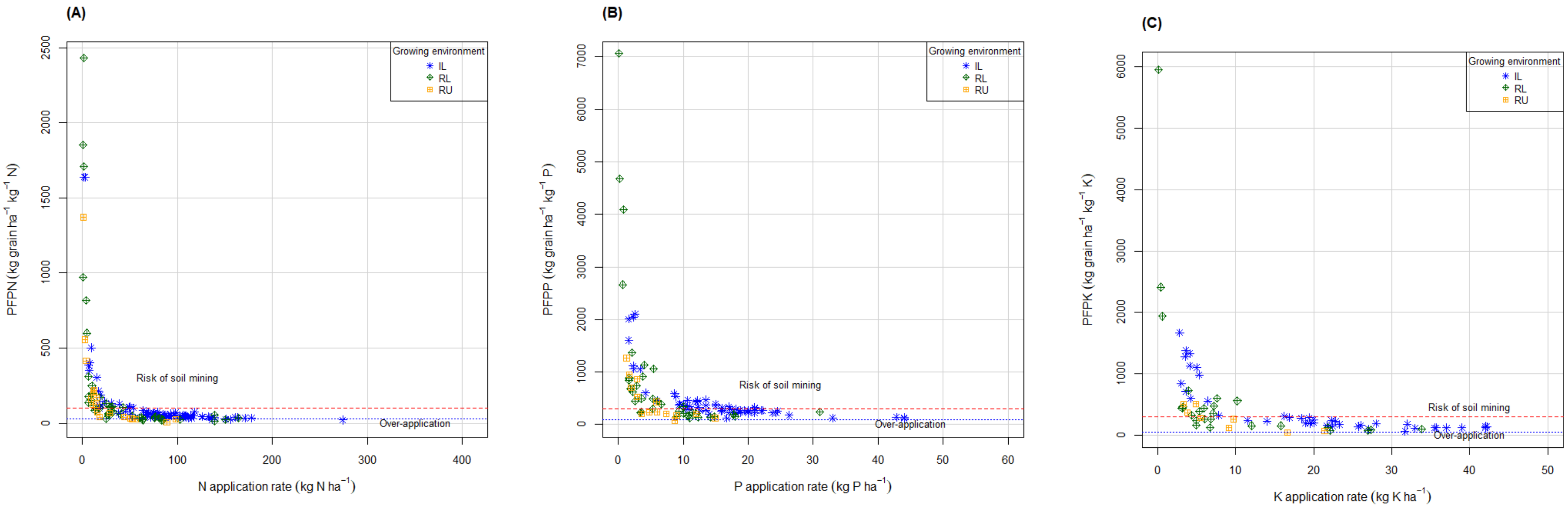

Fig. S2. Partial factor productivity (PFP) of (A) nitrogen (PFPN)- (B) phosphorus (PFPP)- and (C) potassium (PFPK) from data points in irrigated lowland (IL), rainfed lowland (RL) and, rainfed upland (RU) retrieved from surveys/studies conducted in sub-Saharan Africa between 1995 and 2020. The horizontal upper red dashed lines indicate the range of risk of soil mining for the corresponding nutrient. The horizontal lower blue dotted lines indicate the range of possible over-application for the corresponding nutrient.

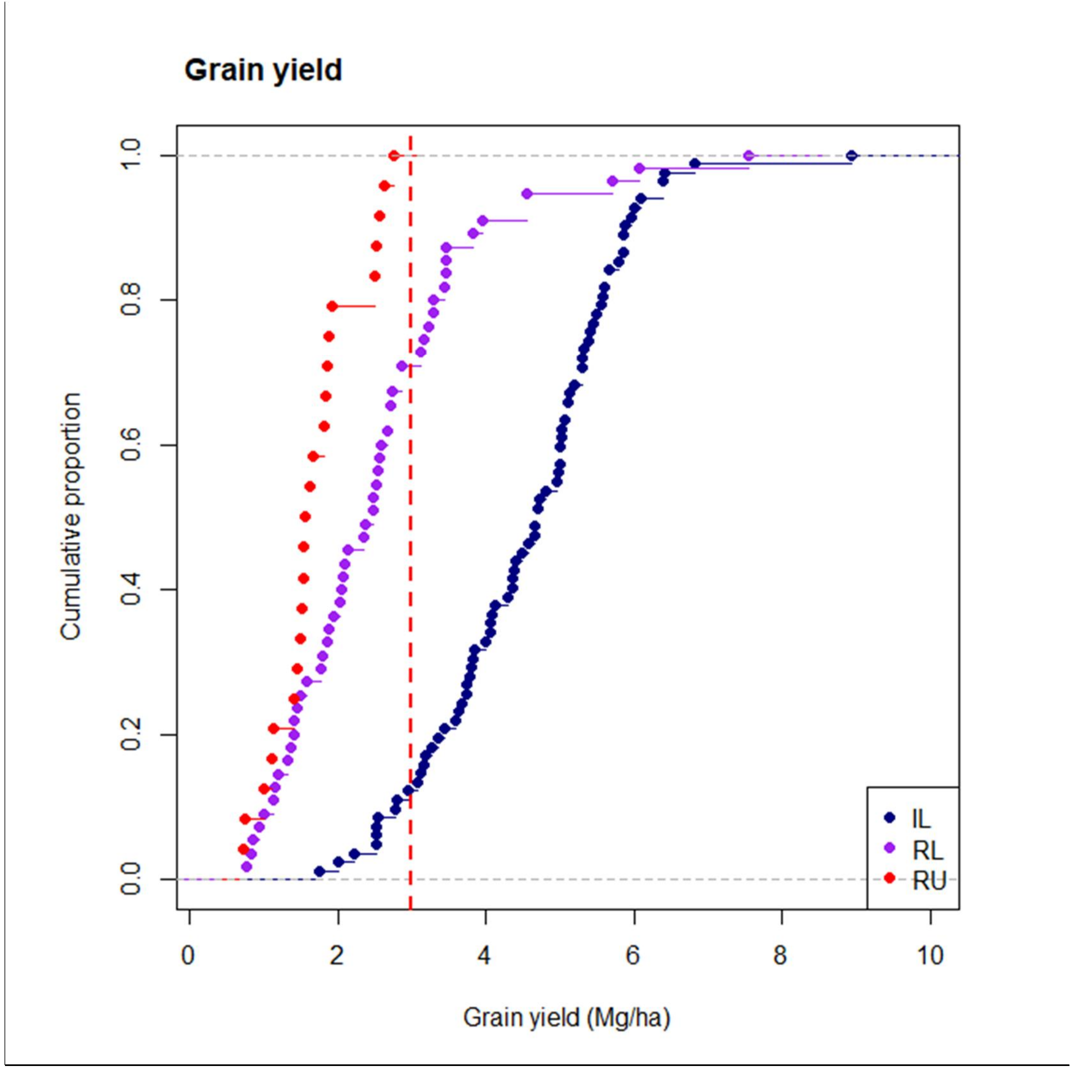

Fig. S3. Cumulative distribution probability of grain yield (Mg ha^{-1}) from data points in irrigated lowland (IL), rainfed lowland (RL), and, rainfed upland (RU) retrieved from surveys/studies conducted in sub-Saharan Africa between 1995 and 2020. The dashed red line represents the global average yield (2.98 Mg ha^{-1}).

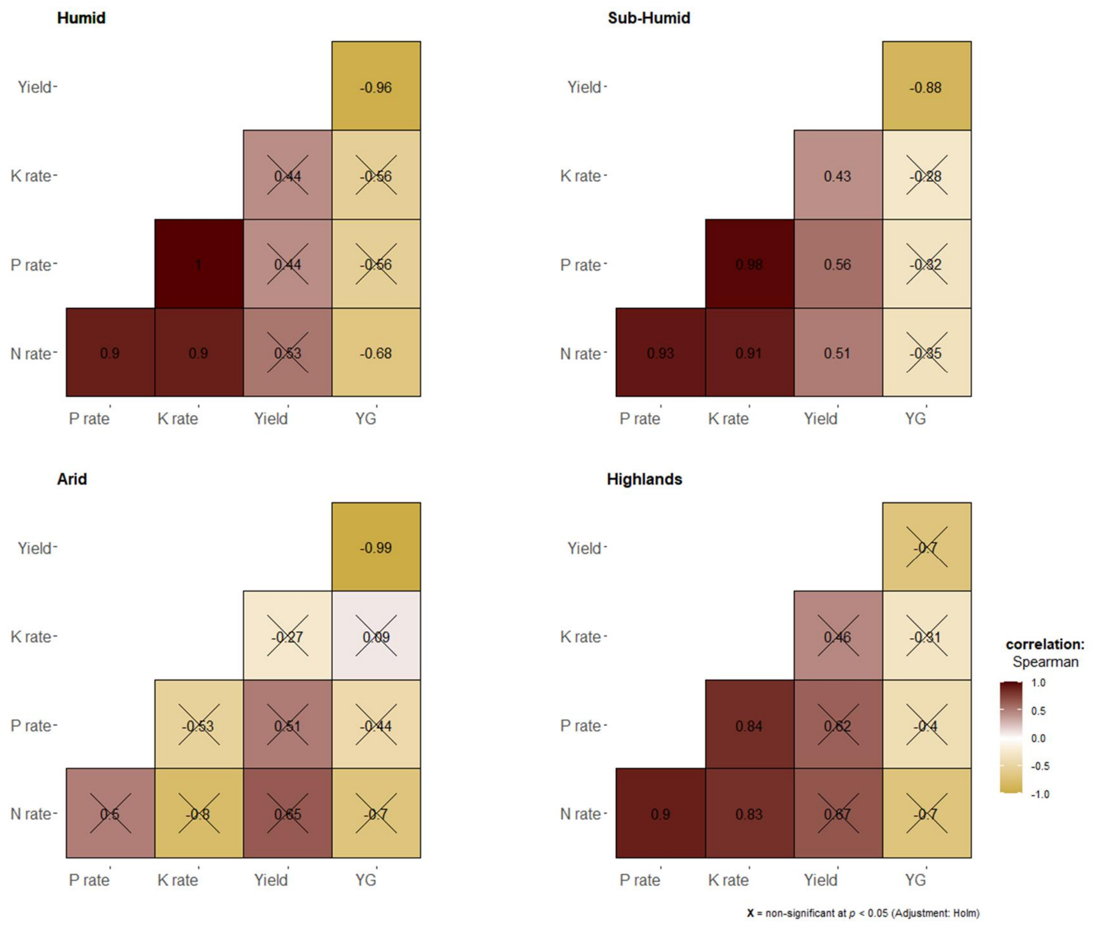

Fig. S4. Correlogram showing the relationships between nutrients (N, P, and K) application rates, rice grain yield, and relative yield gap in different studies across growing environments [irrigated lowland (IL), rainfed lowland (RL) and, rainfed upland (RU)] in sub-Saharan Africa. The values displayed in the matrix are Spearman's rank correlation coefficients (ρ). Non-significant ρ ($p < 0.05$) are marked with an X. Positive correlations are displayed in maroon (or dark red) and negative correlations in goldenrod. The color intensity (see color bar) of the square is proportional to the correlation coefficient.

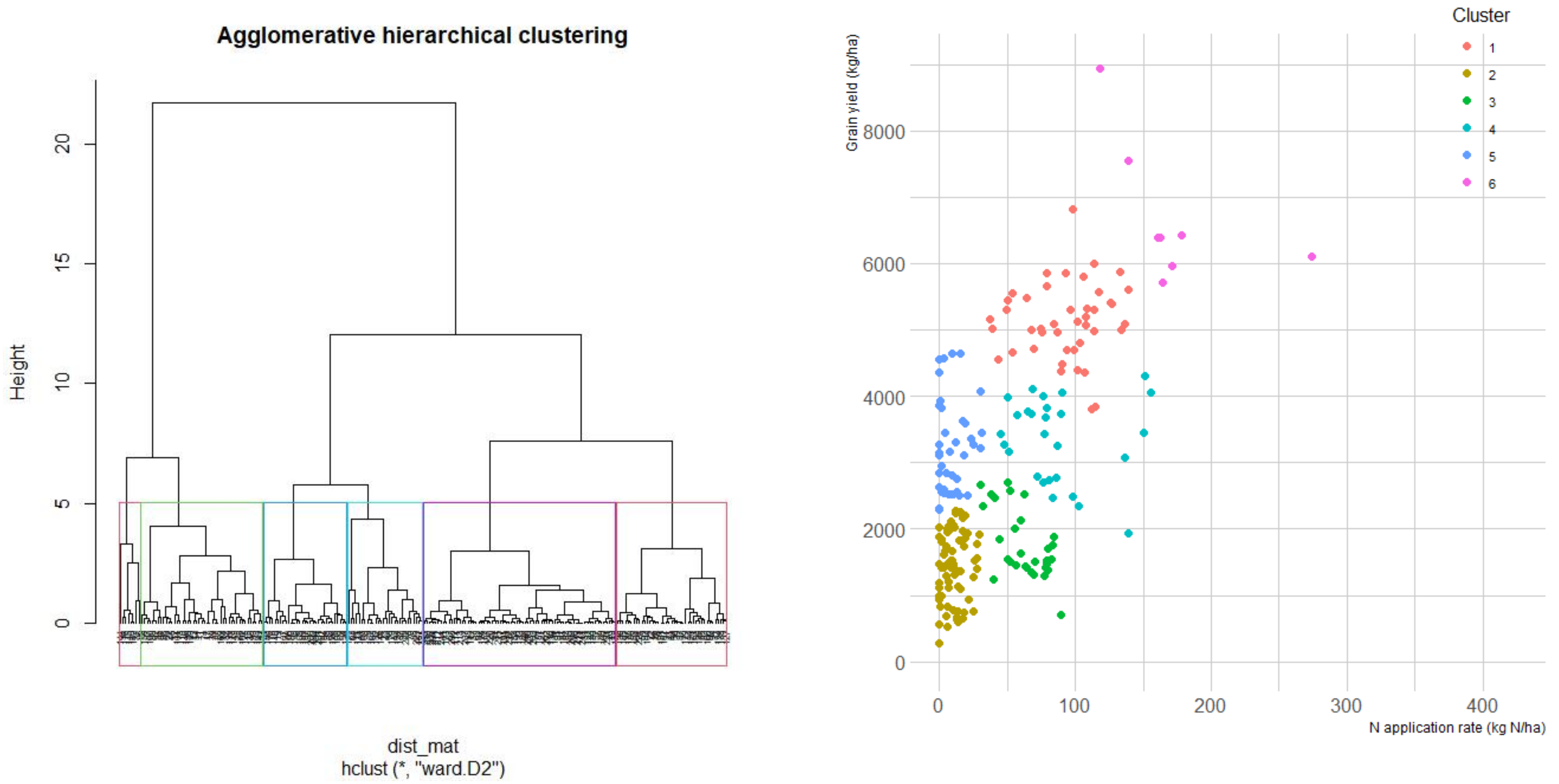

Fig. S5. Agglomerative hierarchical cluster analysis on 224 data points (from studies conducted between 1995 and 2020 in sub-Saharan Africa) using grain yield (kg/ha) and N application rate (kg N/ha). Colored boxes and points describe the 6 clusters or groups:

Cluster 1: High Yield and High N application rate (HYHN);

Cluster 2: Low Yield and Low N application rate (LYLN);

Cluster 3: Low Yield and Medium N application rate (LYMN);

Cluster 4: Medium Yield and High N application rate (MYHN);

Cluster 5: Medium Yield and Low N application rate (MYLN);

Cluster 6: Very High Yield and Very High N application rate (VHYVHN)

References of the studies included the systematic literature review

(Africa Rice Center (AfricaRice) and Syngenta Foundation, 2016; Arouna et al., 2021; Becker et al., 2003; Dossou-Yovo et al., 2020; Dossou-Yovo and Saito, 2021; FAO, 2021; Ibrahim et al., 2022; IFA/IFDC/IPI/PPI/FAO, 2002; Kihoro et al., 2013; Komatsu et al., 2022; Ludemann et al., 2022; Ministère de l'Agriculture et des Aménagements Hydro-agricoles (MAAH), 2021; Muhunyu, 2012; Nakano et al., 2011; Ozaki and Sakurai, 2021; Saito et al., 2015; Senthilkumar et al., 2020; Tanaka et al., 2015, 2013; Tsujimoto et al., 2019, 2014; Wopereis et al., 1999; World Bank, 2021)

1. Africa Rice Center (AfricaRice), Syngenta Foundation, 2016. Report on Calibrating and Validating an ICT-based Decision Support System for Nutrient Management for Rice in West Africa. Africa Rice Center, Cotonou, Benin.
2. Arouna, A., Devkota, K.P., Yergo, W.G., Saito, K., Frimpong, B.N., Adegbola, P.Y., Depieu, M.E., Kenyi, D.M., Ibro, G., Fall, A.A., Usman, S., 2021. Assessing rice production sustainability performance indicators and their gaps in twelve sub-Saharan African countries. Field Crops Research 271, 108263. https://doi.org/10.1016/j.fcr.2021.108263
3. Becker, M., Johnson, D.E., Wopereis, M.C.S., Sow, A., 2003. Rice yield gaps in irrigated systems along an agro-ecological gradient in West Africa. J. Plant Nutr. Soil Sci. 166, 61–67. https://doi.org/10.1002/jpln.200390013
4. Dossou-Yovo, E.R., Saito, K., 2021. Impact of management practices on weed infestation, water productivity, rice yield and grain quality in irrigated systems in Côte d'Ivoire. Field Crops Research 270, 108209. https://doi.org/10.1016/j.fcr.2021.108209
5. Dossou-Yovo, E.R., Vandamme, E., Dieng, I., Johnson, J.-M., Saito, K., 2020. Decomposing rice yield gaps into efficiency, resource and technology yield gaps in sub-Saharan Africa. Field Crops Research 258, 107963. https://doi.org/10.1016/j.fcr.2020.107963
6. FAO, 2021. FAOSTAT statistical database. http://www.fao.org/faostat/en/#data/QCL (accessed 9.21.21).
7. Ibrahim, A., Saito, K., Kokou, A., Johnson, J.-M., Diagne, M., Fagnombo, D.J., Felix, F., Sylvia, B.O., Martial, H., 2022. Seizing opportunity towards sustainable rice cultivation in sub-Saharan Africa. Environmental and Sustainability Indicators 15, 100189. https://doi.org/10.1016/j.indic.2022.100189
8. IFA/IFDC/IPI/PPI/FAO, 2002. Fertilizer use by crop. Rome, Italy.
9. Kihoro, J., Bosco, N.J., Murage, H., Ateka, E., Makihara, D., 2013. Investigating the impact of rice blast disease on the livelihood of the local farmers in greater Mwea region of Kenya. SpringerPlus 2, 308. https://doi.org/10.1186/2193-1801-2-308
10. Komatsu, S., Saito, K., Sakurai, T., 2022. Changes in production, yields, and the cropped area of lowland rice over the last 20 years and factors affecting their variations in Côte d′Ivoire. Field Crops Research 277, 108424. https://doi.org/10.1016/j.fcr.2021.108424
11. Ludemann, C.I., Gruere, A., Heffer, P., Dobermann, A., 2022. Global data on fertilizer use by crop and by country. Sci Data 9, 501. https://doi.org/10.1038/s41597-022-01592-z

12. Ministère de l'Agriculture et des Aménagements Hydro-agricoles (MAAH), 2021. Tableau de bord statistique de l'Agriculture 2020. Ministère de l'Agriculture et des Aménagements Hydro-agricoles (MAAH), Ouagadougou, Burkina Faso.
13. Muhunyu, J.G., 2012. Is doubling rice production in Kenya by 2018 achievable? Journal of Developments in Sustainable Agriculture 7, 46–54. https://doi.org/10.11178/jdsa.7.46
14. Nakano, Y., Bamba, B., Diagne, A., Otsuka, K., Kajisa, K., 2011. The Possibility of a Rice Green Revolution in Large-scale Irrigation Schemes in Sub-Saharan Africa, Policy Research Working Papers. The World Bank. https://doi.org/10.1596/1813-9450-5560
15. Ozaki, R., Sakurai, T., 2021. Profitability of chemical fertilizer application：Comparison of lowland and upland rice cultivation in Madagascar. JJAE 23, 119–124. https://doi.org/10.18480/jjae.23.0_119
16. Saito, K., Diack, S., Dieng, I., N'Diaye, M.K., 2015. On-farm testing of a nutrient management decision-support tool for rice in the Senegal River valley. Computers and Electronics in Agriculture 116, 36–44. https://doi.org/10.1016/j.compag.2015.06.008
17. Senthilkumar, K., Rodenburg, J., Dieng, I., Vandamme, E., Sillo, F.S., Johnson, J. -M., Rajaona, A., Ramarolahy, J.A., Gasore, R., Abera, B.B., Kajiru, G.J., Mghase, J., Lamo, J., Rabeson, R., Saito, K., 2020. Quantifying rice yield gaps and their causes in Eastern and Southern Africa. J Agro Crop Sci 206, 478–490. https://doi.org/10.1111/jac.12417
18. Tanaka, A., Diagne, M., Saito, K., 2015. Causes of yield stagnation in irrigated lowland rice systems in the Senegal River Valley: Application of dichotomous decision tree analysis. Field Crops Research 176, 99–107. https://doi.org/10.1016/j.fcr.2015.02.020
19. Tanaka, A., Saito, K., Azoma, K., Kobayashi, K., 2013. Factors affecting variation in farm yields of irrigated lowland rice in southern-central Benin. European Journal of Agronomy 44, 46–53. https://doi.org/10.1016/j.eja.2012.08.002
20. Tsujimoto, Y., Muranaka, S., Saito, K., Asai, H., 2014. Limited Si-nutrient status of rice plants in relation to plant-available Si of soils, nitrogen fertilizer application, and rice-growing environments across Sub-Saharan Africa. Field Crops Research 155, 1–9. https://doi.org/10.1016/j.fcr.2013.10.003
21. Tsujimoto, Y., Rakotoson, T., Tanaka, A., Saito, K., 2019. Challenges and opportunities for improving N use efficiency for rice production in sub-Saharan Africa. Plant Production Science 1–15. https://doi.org/10.1080/1343943X.2019.1617638
22. Wopereis, M.C.S., Donovan, C., NebieÂ, B., Guindo, D., N'Diaye, M.K., 1999. Soil fertility management in irrigated rice systems in the Sahel and Savanna regions of West Africa Part I. Agronomic analysis. Field Crops Research 61, 125–145. https://doi.org/10.1016/S0378-4290(98)00154-3
23. World Bank, 2021. Microdata Library: Living Standards Measurements Study. https://microdata.worldbank.org/index.php/catalog/lsms (accessed 1.10.21).
